# Comparable neuroprotection efficacy of raw Pu-erh tea and ripened Pu-erh tea in D-galactose-induced aging mice via gut-brain axis

**DOI:** 10.1038/s41538-026-00872-x

**Published:** 2026-05-05

**Authors:** Liyuan Peng, Hongzhe Zeng, Xiaomei Yang, Liwei Wan, Qixian Bai, Linmei Liu, Hui Rao, Hui Li, Xingrui Xiong, Linlin Li, Jiuyun Chu, Weitao Wang, Songtao Pu, Jian’an Huang, Zhonghua Liu

**Affiliations:** 1 Key Laboratory of Tea Science of the Ministry of Education, Hunan Agricultural University, Changsha, China; 2 National Research Center of Engineering and Technology for Utilization of Botanical Functional Ingredients Changsha, Changsha, China; 3 Yuelushan Laboratory, Changsha, China; 4 National Key Laboratory for Tea Plant Germplasm Innovation and Resource Utilization, Changsha, China; 5 Key Laboratory for Evaluation and Utilization of Gene Resources of Horticultural Crops, Ministry of Agriculture and Rural Affairs of China, Hunan Agricultural University, Changsha, China; 6 Yunnan Xiaguantuo Tea (Group) Corporation Company Limited Yunnan, Dali, China

**Keywords:** Diseases, Microbiology, Neurology, Neuroscience

## Abstract

Prevention of age-related cognitive decline by tea consumption is of great interest. This study systematically compared the neuroprotective efficacy of raw Pu-erh tea (RPT) and ripened Pu-erh tea (FPT) against D-galactose-induced aging in mice, focusing on the modulation of the gut-brain axis. To enhance translational relevance, mice were provided with ad libitum access to RPT or FPT infusions, mimicking human drinking habits. Results showed that both RPT and FPT significantly ameliorated cognitive impairment and hippocampal damage in aging mice, with comparable efficacy despite their distinct phytochemical profiles. Both teas reversed gut microbiota dysbiosis, consistently enriching core taxa such as *Lachnospiraceae_NK4A136_group* and *Alistipes*, and restored host sphingolipid metabolism, leading to reduced cerebral ceramide levels and A*β* deposition. Notably, the key difference lay in polyphenol components: RPF acted mainly via native monomeric catechins, whereas FPT relied on fermentation-derived polymers (theaflavins, thearubigins, theabrownins) and gallic acid. Despite fundamental compositional differences imposed by pile fermentation, both teas provided similar protection against age-related cognitive decline, primarily through the gut microbiota–sphingolipid–brain axis. Our findings highlight that both RPT and FPT represent effective dietary interventions for cognitive health, with the choice being a matter of preference.

## Introduction

The aging of the global population has evolved into an irreversible demographic trend. As documented in statistics released by the United Nations Population Division, the number of people aged 60 years and over worldwide is anticipated to exceed 2.1 billion by 2050. In China, this aging process is even more pronounced, with the proportion of the population aged 60 and above projected to surpass 30% in the same timeframe. This profound transformation in population structure is imposing unprecedented challenges on public health systems globally. Population aging is invariably associated with a heightened incidence of chronic diseases, among which cancer, cardiovascular diseases, and neurodegenerative diseases stand as the three prominent disease categories endangering the health of the elderly population^1^. Among these, neurodegenerative diseases—marked by prolonged disease progression, unfavorable prognosis, and most notably, concomitant cognitive impairment—exert a substantial adverse impact on both the lifespan and health-related quality of life of elderly individuals^2^. As such, they have emerged as a critical public health issue of widespread concern in the context of global population aging.

In recent years, natural products with a long history of human consumption have garnered increasing attention for their potential in modulating aging and aging-related diseases, owing to their favorable safety profiles and high acceptability. As a globally prevalent beverage, tea has been linked to impacts on the aging process and aging-related diseases, with this connection supported by data from epidemiological studies, clinical observations, and experiments utilizing various animal models^3–5^. However, existing research on tea’s anti-aging and cognitive-protective effects still has notable gaps that limit its translational relevance to real-world scenarios.

A key unresolved issue is which type of tea, raw Pu-erh tea or ripened Pu-erh tea, possesses superior anti-aging efficacy—a question of significant public and consumer interest. While both are widely consumed for their purported health benefits, whether their neuroprotective efficacy meaningfully diverges remains to be elucidated. This uncertainty stems from the fundamental alteration of phytochemical profiles driven by their distinct processing, specifically pile fermentation. This process converts native catechins in raw tea into compounds such as theabrownins via microbial activity, potentially leading to divergent effects on aging-related pathways. Nevertheless, direct comparisons between these tea types are lacking, resulting in an oversimplified understanding that hinders the development of tailored interventions.

Another notable limitation is that existing tea intervention studies commonly rely on gavage administration of high-dose tea extracts, which markedly deviates from human consumption patterns and compromises translational relevance. First, the decoction process used to prepare gavage extracts alters tea’s natural bioactivity^6^, creating a composition distinct from daily tea infusions. Second, gavage induces non-physiological “peak-valley” concentration fluctuations, unlike the stable levels maintained through habitual tea consumption^7^. Additionally, the invasive nature of gavage triggers stress responses that may confound metabolic readouts^8^, while bypassing oral processing—an essential step in human tea digestion that modulates component bioavailability and bioactivity^9^. These factors collectively limit the ecological validity of gavage-based models.

Critically, the gut-brain axis is widely recognized as a crucial pathway linking gut microbiota to brain function, and dietary polyphenols—including those found in tea—are known modulators of gut microbial composition^10–12^. However, translational evidence directly connecting tea-mediated gut microbiota changes to measurable cognitive benefits remains limited. Therefore, systematic studies are still needed to compare the regulatory effects of different tea types on the gut-brain axis and to evaluate their corresponding influences on cognitive function.

Therefore, the present study was designed with four core objectives: (1) to systematically compare the efficacy of RPT and FPT in ameliorating cognitive impairment in aged mice, clarifying their differential effects on age-related cognitive decline; (2) to allow animals free access to tea infusion (mimicking daily human tea consumption), thereby ensuring the ecological validity of the findings and their alignment with real-world tea consumption patterns; (3) to investigate the role of the gut-brain axis in mediating tea’s cognitive-protective effects; (4) to identify core cognitive-improving components in RPT and FPT. By addressing these objectives, this research seeks to provide favorable insights into the application of tea as a safe, accessible strategy for mitigating age-related cognitive decline.

## Results

### Effects of voluntary RPT/FPT consumption on basic physiological indicators in AG mice

**Fig. 1:**
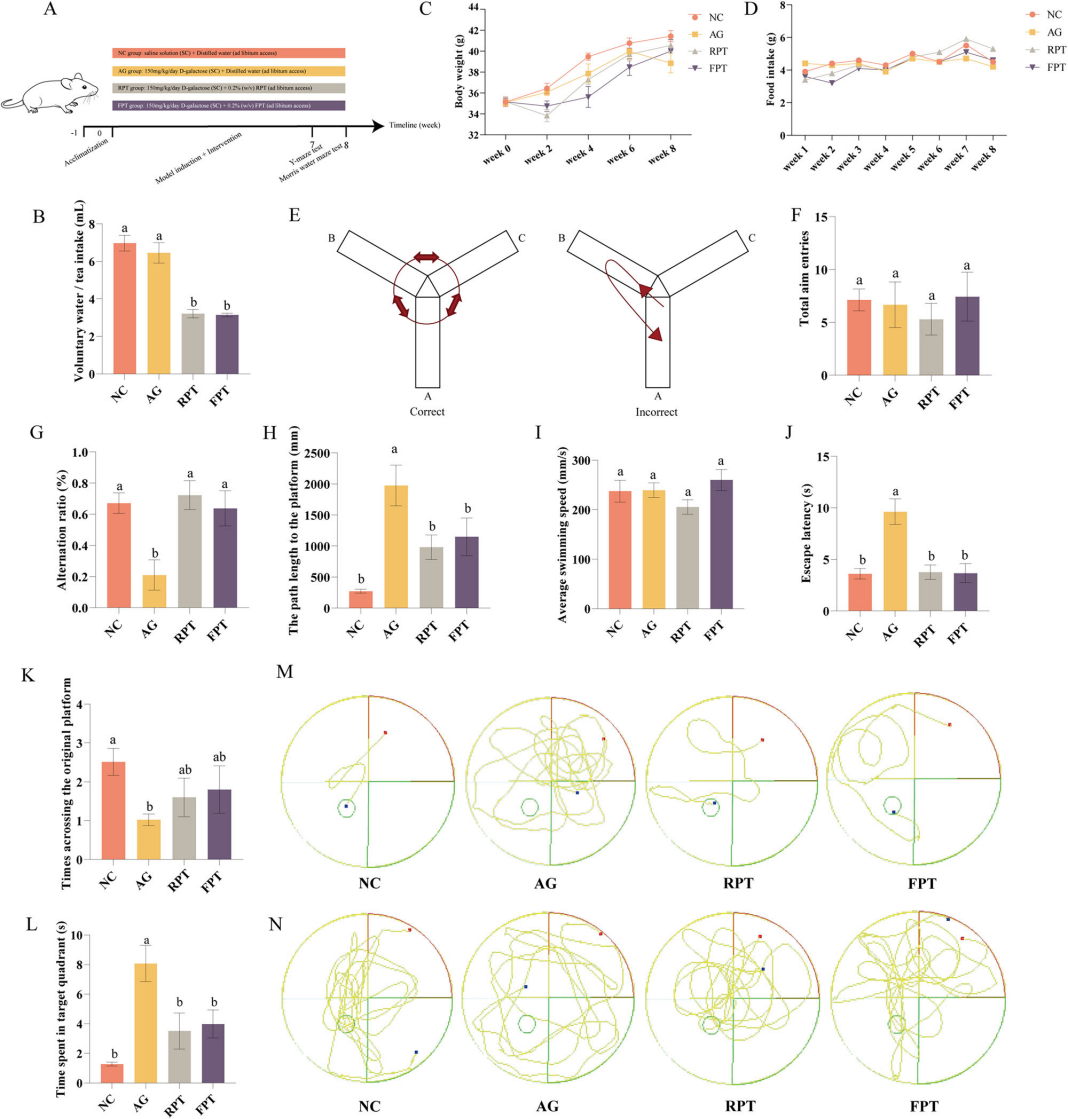
Effects of RPT/FPT on D-galactose-induced cognitive deficits in mice. **A** Schematic of the treatment protocol; **B** voluntary water/tea intake; **C** body weight; **D** average food intake; **E** the path length to the platform; **F** Y-maze spontaneous alternation schematic; **G** total arm entries; **H** alternation ratio; **I** average swimming speed; **J** escape latency; **K** times crossing the original platform; **L** time spent in the target quadrant; **M** goal-directed navigation trajectory map; **N** platform crossing frequency map, Data are presented as the mean ± SEM (*n* = 8). Different lowercase letters (a, b, c, …) above the bars indicate statistically significant differences (*P* < 0.05) as determined by one-way ANOVA followed by Fisher’s LSD post hoc test. Groups that do not share a common letter are significantly different from each other. the same as below.

To evaluate the impact of free access to RPT/FPT on the basic physiological signs of mice subjected to D-galactose modeling, we continuously monitored the voluntary water/tea consumption, body weight, and food intake of the mice. As shown in Fig. 1B, significant differences in voluntary fluid intake were observed among the groups. Mice in the NC and AG groups (provided with water) exhibited a higher average daily intake of approximately 7 mL per mouse. In contrast, voluntary intake was significantly lower in the RPT and FPT groups (provided with tea), averaging around 3.0 mL per mouse per day. This difference could be attributed to an inherent preference for water over tea in mice. Notably, the average daily tea consumption did not differ significantly between the RPT and FPT groups. This observation validates the subsequent comparative analysis of their respective anti-aging efficacies, as the potential differential effects can be attributed to the intrinsic properties of the teas rather than variations in intake volume. With respect to body weight and food intake (Fig. 1C, D), all groups showed an overall increase in body weight over the 8-week period, while food intake fluctuated slightly across groups. No statistically significant differences were detected in body weight among the NC, AG, RPT, and FPT groups. Collectively, these findings indicate that D-galactose modeling and free access to RPT or FPT tea did not exert a significant influence on the long-term body weight gain or food consumption patterns of the mice.

### Effects of RPT/FPT on cognitive impairment in D-galactose-induced AG mice

To investigate the effects of RPT/FPT on cognitive impairment in D-galactose-induced aging mice, the Y-maze test and Morris water maze test were employed to evaluate short-term spatial memory and long-term spatial learning and memory, respectively. Specifically, the Y-maze spontaneous alternation test assesses mice’s short-term memory by quantifying their tendency to explore novel arms based on their memory of previously visited ones. Thus, mice with cognitive impairment tend to make errors in exploring novel arms (Fig. 1E). The results of the Y-maze spontaneous alternation test are presented in Fig. 1F, G. As shown, there was no statistically significant difference in the total number of arm entries among the four groups, indicating that the experimental interventions did not affect the general locomotor activity or exploratory motivation of the mice. In contrast, significant differences were observed in the spontaneous alternation ratio across groups. Specifically, compared with the NC group, the alternation ratio of the AG group was significantly decreased, suggesting impaired short-term spatial memory in aging mice with cognitive deficits. Notably, intervention with RPT or FPT effectively reversed this reduction in the alternation ratio, with no statistically significant difference observed between the RPT and FPT groups. This suggests that both RPT and FPT exert potential protective effects on short-term spatial memory in D-galactose-induced aging mice, with comparable efficacy. For the Morris water maze test, to assess long-term spatial learning and memory, we analyzed key behavioral parameters, including the path length to the platform (Fig. 1H, M), average swimming speed (Fig. 1I), escape latency (Fig. 1J), times crossing the original platform (Fig. 1K), and time spent in the target quadrant (Fig. 1L). As shown, there was no significant difference in average swimming speed among the groups, indicating that experimental interventions didn’t affect mice’s swimming ability. Compared with the NC group, the AG group had a significantly longer path length to the platform and escape latency, and shorter time spent in the target quadrant and fewer times crossing the original platform, suggesting impaired long-term spatial learning and memory in D-galactose-induced aging mice. Notably, RPT and FPT interventions effectively ameliorated these deficits. Both groups showed reduced path length to the platform and escape latency, alongside increased time in the target quadrant and platform crossing times, with all indicators approaching the levels of the NC group. Surprisingly, there were no significant differences in any of the water maze indicators between the RPT group and the FPT group, indicating comparable efficacy of the two interventions in restoring long-term spatial cognitive function. Worthy of note, although previous studies linked increased target quadrant time to memory recovery, our study found decreased time in both the normal control and tea-treated groups^13^. We hypothesize this relates to optimized spatial search strategies—normal mice shift from “edge search” to “focused search” after platform removal, while aging mice show random search. RPT and FPT interventions restored efficient search, reflected by reduced target quadrant time. Visual evidence from the mice’s platform crossing frequency trajectories provides support for this hypothesis (Fig. 1N). Collectively, results from the Y-maze and Morris water maze tests demonstrate that both RPT and FPT effectively ameliorate short-term and long-term spatial cognitive impairments in D-galactose-induced aging mice, with no significant difference in their protective efficacy.

### Effects of RPT/FPT on brain oxidative stress and hippocampus histopathological changes in AG mice

Building upon the behavioral findings that D-galactose and RPT/FPT interventions impacted mice’s spatial learning and memory, and given the intimate link between the brain (particularly the hippocampus) and spatial cognitive abilities^14^, as well as the established mechanism where D-galactose-induced modeling drives oxidative stress through heightened metabolism and excessive free radicals, thereby triggering pathological alterations^15^, we proceeded to explore the effects of RPT/FPT on brain oxidative stress and hippocampal histopathological changes in AG mice.

**Fig. 2:**
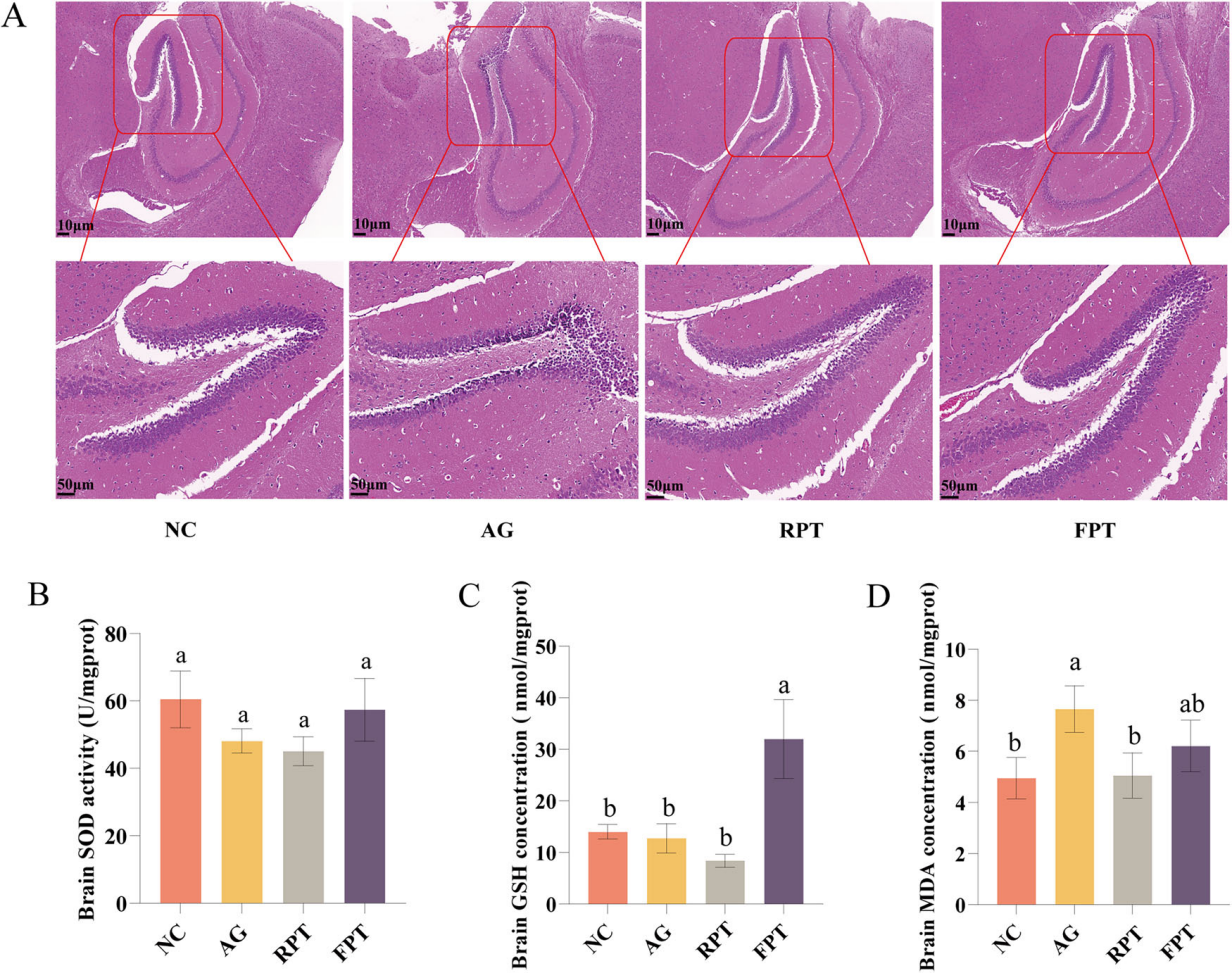
Effects of RPT/FPT on brain oxidative stress and hippocampus histopathological changes in AG mice. **A** Representative H&E staining images of hippocampus histopathological changes; **B** brain SOD activity; **C** brain GSH concentration; **D** brain MDA concentration.

As shown in Fig. 2A, representative hematoxylin and eosin (H&E) staining images revealed distinct histopathological changes in the hippocampus across groups. In the NC group, the hippocampal tissue structure was intact, with neatly arranged cells and clear boundaries. In contrast, the AG group exhibited obvious hippocampal damage, characterized by blurred contours of the dentate gyrus, obscured serrated structures, disordered arrangement of granular cell layer cells with disrupted normal single-layered and orderly pattern, loss of cell polarity, and ectopic migration of some cells to the molecular layer. A substantial reduction in the number of neurons was observed in the polymorphic layer, along with cell body shrinkage, nuclear pyknosis, and hyperchromasia, which are typical morphological features of apoptosis^16^. Additionally, there was an increase in vacuolation of cells in the principal cell layer and enlarged intercellular spaces. Notably, RPT and FPT interventions alleviated these pathological alterations; the hippocampal tissue in both RPT and FPT groups showed improved cell arrangement and reduced degeneration, approaching the histological features of the NC group. Regarding brain oxidative stress indicators, as depicted in Fig.2B–D, no statistically significant difference in SOD activity was observed among the four groups, indicating that neither D-galactose-induced aging nor RPT/FPT intervention affected the activity of this key antioxidant enzyme. For GSH, D-galactose modeling did not alter its brain levels, while FPT intervention significantly elevated GSH concentration compared to the AG group. As for MDA concentration, a marker of lipid peroxidation, the AG group displayed a significant increase compared to the NC group (*P* < 0.05). Both RPT and FPT treatments reduced MDA concentration, with values closer to those of the NC group, though the regulatory effect of FPT on MDA was slightly more pronounced (no statistical difference from RPT). Collectively, these results demonstrated that RPT and FPT could alleviate D-galactose-induced brain oxidative damage in AG mice, and FPT specifically exerted a superior regulatory effect on GSH, which might contribute to the improvement of hippocampal histopathological damage.

### Effects of RPT/FPT on gut microbiota structure in AG mice

**Fig. 3:**
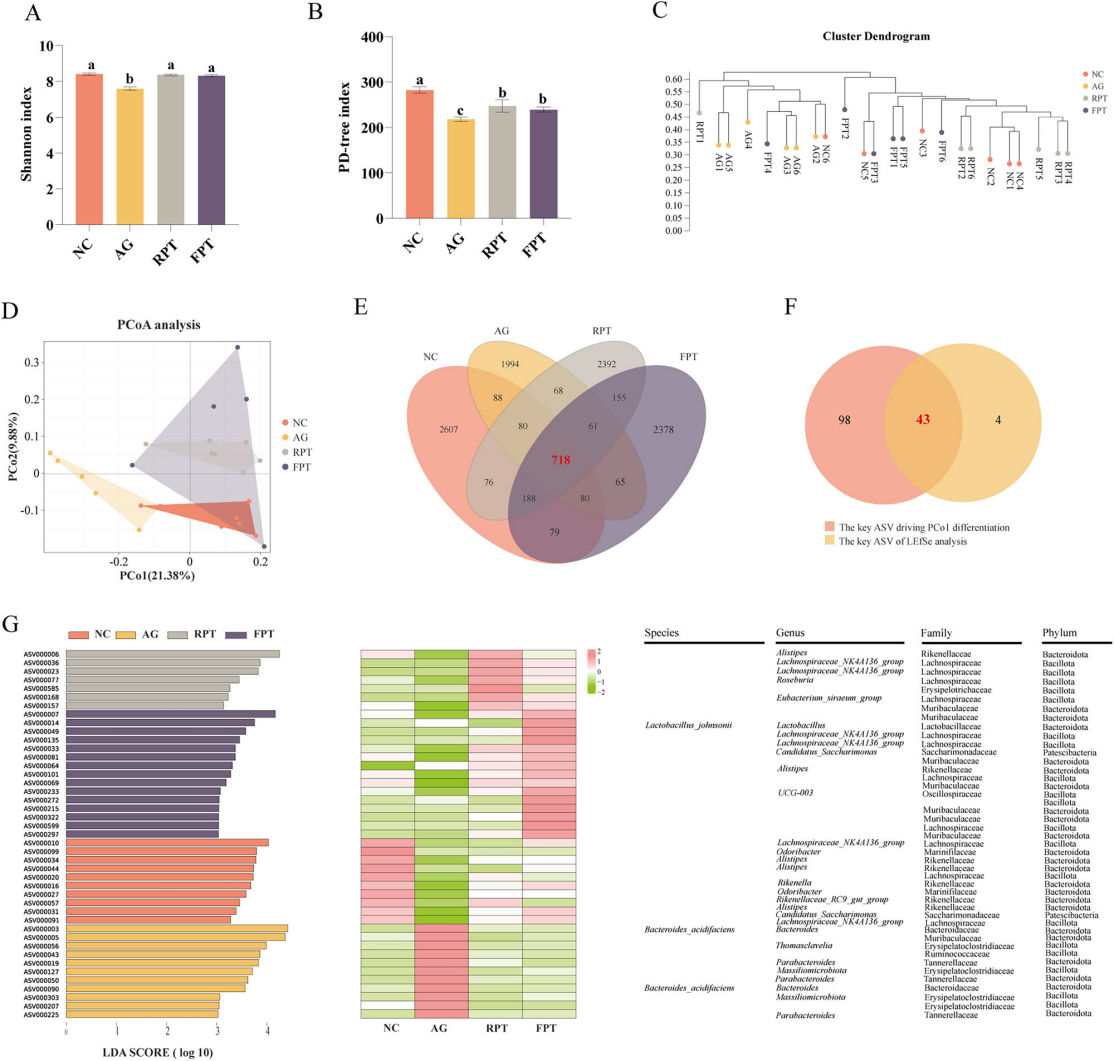
RPT/FPT interventions effectively reverse AG-induced gut dysbiosis by reinstating NC-like microbial profiles. **A** Shannon index; **B** PD-tree index; **C** cluster dendrogram; **D** PCoA analysis; **E** 718 Overlapped ASVs across all groups; **F** 43 key ASVs identified by both LEfSe analysis and PCo1 differentiation; **G** LEfSe results of 43 Key ASVs.

To investigate the influence of tea-gut microbiota interactions on the gut microbiota features that underlie the backgrounds in AG mice, we performed 16S rRNA sequencing on collected mouse feces and compared the gut microbial characteristics in AG mice. The Shannon and PD-tree indexes are used to assess the species evenness and lineage diversity of the microbial communities in this study. As shown in Fig. 3A, B, the Shannon and PD-tree indices of intestinal microbiota were significantly reduced in D-galactose-induced (intraperitoneal injection) AG mice compared to normal mice (*P* < 0.05). Notably, these two diversity indices increased markedly following RPT and FPT intervention (*P* < 0.05), indicating effective restoration of gut dysbiosis. Since all mice received the same diet, dietary interference was excluded. These findings confirm that cognitive impairment pathology is connected with gut dysbiosis and that RPT/FPT intervention distinctly ameliorates this imbalance. To further visualize differences in the overall structure of intestinal microbial communities among groups, we performed UPGMA clustering analysis (Fig. 3C). Results showed that AG mice clustered tightly into a separate branch, whereas samples from the RPT and FPT intervention groups clustered interspersedly with normal mice. These findings indicate that the pathological state of cognitive impairment accompanies a characteristic shift in the intestinal microbial community, and RPT/FPT intervention effectively reverses this shift, restoring the overall microbial structure to a normal state. Principal coordinate analysis (PCoA) based on Bray-Curtis distances further confirmed the inter-group separation pattern of the gut microbial communities. Along the PCo1 axis, the AG group was significantly separated from all other groups, while the tea intervention groups (RPT and FPT) showed substantial overlap with the normal control group along both PCo1 and PCo2, with no distinct boundaries between them. This visual separation pattern was supported by statistical testing: permutational multivariate analysis of variance (PERMANOVA) confirmed highly significant differences in community structure among groups (*R*² = 0.313, *P* = 0.02), and a test for homogeneity of multivariate dispersions (PERMDISP) verified that within-group dispersion was homogeneous across groups (*P* = 0.0599), thereby ruling out differences in within-group variation as the driver of the observed pattern (Fig. S1A, B). Consistent with UPGMA clustering results, these findings verify that cognitive impairment accompanies characteristic differentiation of the microbial community, and that RPT/FPT intervention effectively reverses this trend.

### Uncovering the differential taxa impacted by RPT/FPT in AG mice

To dissect the structural remodeling of gut microbiota induced by RPT/FPT intervention in AG mice, we analyzed differentially abundant bacterial taxa across experimental groups. Gut microbial profiles at the phylum, family, and genus taxonomic levels are illustrated in Fig. S1C–E, which revealed that mice with cognitive impairment exhibit taxonomic-specific dysbiosis. Amplicon Sequence Variants (ASVs), clustered with 100% sequence similarity, offer representation of unique, naturally occurring biological sequence variants. To avoid “information blurring” inherent to higher taxonomic levels, we performed LEfSe analysis at the ASV level. First, a Venn diagram identified 718 ASVs common to all groups—these stably occurring taxa allowed exclusion of random “presence-absence” variations stemming from technical bias or sample heterogeneity. Second, LEfSe analysis (LDA > 3) on these 718 shared ASVs identified 47 ASVs as differentially abundant across the NC, AG, RPT, and FPT groups (Fig. S2). However, some of the predominant ASVs were classified into the same cluster, suggesting that the scope of key microorganisms should be further defined.

As previously noted, the AG group was significantly separated from all other groups along PCo1. We thus conducted correlation analysis between the 718 shared ASVs and PCo1 scores of all samples, identifying 141 ASVs that may contribute to the separation between the AG group and other intervention groups. To narrow down the scope of key microorganisms (as prompted by the clustering of some predominant ASVs), we took the intersection of these 47 differential ASVs and 141 separation-related ASVs, ultimately obtaining 43 common ASVs. LEfSe analysis results for these refined core ASVs are depicted in Fig. 3G, which clearly delineates taxonomic biomarkers that distinguish the NC, AG, RPT, and FPT groups. Specifically, the AG group was characterized by significant enrichment of *Bacteroides acidifaciens*, along with the genera *Bacteroides*, *Thomasclavelia*, *Parabacteroides*, and *Massiliomicrobiota*. In contrast, the RPT group showed a distinct taxonomic profile, with enrichment of *Alistipes*, *Lachnospiraceae_NK4A136_group*, *Roseburia*, and *Eubacterium_siraeum_group*. The FPT intervention drove enrichment of multiple taxa, including *Lactobacillus_johnsonii*, the genus *Lactobacillus*, *Lachnospiraceae_NK4A136_group*, *Candidatus_Saccharimonas*, *Alistipes*, and *UCG-003*. For the NC group, the enriched taxa comprised *Lachnospiraceae_NK4A136_group*, *Odoribacter*, *Alistipes*, *Rikenella*, *Rikenellaceae_RC9_gut_group*, and *Candidatus_Saccharimonas*. Notably, several core ASVs enriched in the RPT or FPT groups clustered within the same taxonomic lineages as those characteristics of the NC group, reflecting a clear convergence toward normal gut microbiota. For example, *Lachnospiraceae_NK4A136_group*, *Alistipes* were consistently enriched across the RPT, FPT, and NC groups (with significantly lower abundance in the AG group), forming a shared biomarker of microbial homeostasis. These LEfSe-derived taxonomic signatures directly support the findings from PD-tree and PCoA analyses. Collectively, this taxonomic and phylogenetic convergence reinforces the conclusion that RPT/FPT interventions effectively reverse AG-induced gut dysbiosis by reinstating NC-like microbial profiles.

### Effects of RPT/FPT intervention on serum metabolic profiling in AG mice

**Fig. 4:**
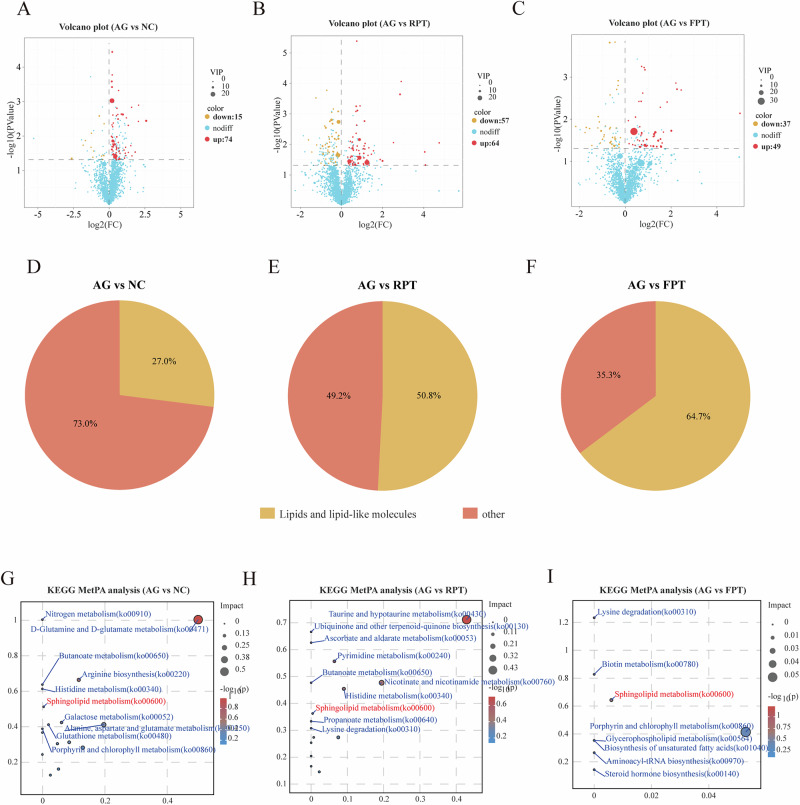
Effects of RPT/FPT intervention on serum metabolic profiling in AG mice. **A** Volcano plot of AG vs NC; **B** volcano plot of AG vs RPT; **C** volcano plot of AG vs FPT; **D** chart of serum DAMs classification between AG and NC groups; **E** chart of serum DAMs classification between AG and RPT groups; **F** chart of serum DAMs classification between AG and FPT groups; **G** KEGG MetPA analysis of AG vs NC; **H** KEGG MetPA analysis of AG vs RPT; **I** KEGG MetPA analysis of AG vs FPT.

The bidirectional communication between the gut microbiota and the central nervous system could be mediated by circulating metabolites. Based on this mechanism, the present study analyzed serum metabolites to systematically characterize the global alterations in metabolic profiles of aged mice following RPT and FPT intervention. Firstly, orthogonal partial least squares discriminant analysis (OPLS-DA) was employed to evaluate the overall separation of serum metabolite profiles between groups. As depicted in Fig. S3A–C, clear separations were observed in OPLS-DA score plots for NC vs AG, AG vs RPT, and AG vs FPT comparisons, with permutation tests validating model reliability. These results indicated that RPT and FPT interventions reshaped the serum metabolite landscape. Subsequently, volcano plots were generated to identify differentially abundant metabolites (DAMs) between groups with the criteria of *P* < 0.05 and VIP > 1^17^. As shown in Fig. 4A–C, compared with the NC group, the AG group had 15 downregulated and 74 upregulated DAMs. For the AG vs RPT comparison, there were 57 downregulated and 64 upregulated DAMs in the RPT group relative to the AG group. In the AG vs FPT comparison, the FPT group showed 37 downregulated and 49 upregulated DAMs compared to the AG group. Furthermore, the KEGG pathway classification histogram revealed that RPT and FPT interventions significantly increased the involvement in lipid metabolism pathways (Fig. S3D). To validate this, we analyzed the classification of DAMs across groups (Supplementary Tables 1 and 2). As shown in Fig. 4D–F, in the AG vs NC comparison, Lipids and lipid-like molecules accounted for 27.0% of DAMs. For AG vs RPT, this category made up 50.8% of DAMs, and in AG vs FPT, it constituted 64.7% of DAMs. These results indicated that RPT and FPT interventions primarily regulated serum lipid metabolites, which might serve as crucial mediators in gut-brain axis communication and cognitive function modulation. To further pinpoint underlying gut-brain axis regulation (and exclude confounding signals from exogenous metabolites, such as dietary residues or environmental contaminants that are not inherently involved in host-microbiota crosstalk), we first excluded non-endogenous differential metabolites by using the Human Metabolome Database. Then, we examined the KEGG MetPA results for AG vs NC (Fig. 4G), AG vs RPT (Fig. 4I), and AG vs FPT (Fig. 4J). Notably, sphingolipid metabolism (ko00600) was the only pathway consistently identified across all three comparisons. This convergence suggests that RPT/FPT may act by restoring sphingolipid metabolism, which in turn modulates host systemic metabolism.

### Identifying key microbial taxa and hippocampal sphingolipid alternations underlying RPT/FPT-mediated cognitive improvement in AG mice

Building on serum metabolomic findings, which highlighted the potential role of sphingolipid metabolism in RPT/FPT-induced cognitive improvement. We sought to gain functional insights at the microbial level that could complement the serum metabolomic findings implicating sphingolipid metabolism. A predictive analysis of gut microbiota functional potential using PICRUSt2 was performed. This in silico prediction based on taxonomic profiles offered a complementary perspective, indicating that pathways including microbially mediated sphingolipid metabolism could potentially be modulated by the RPT and FPT interventions (Fig. S4). However, critical knowledge gaps remain to solidify this mechanistic link. Firstly, it remains unclear whether the microbial taxa driving sphingolipid metabolism restoration are the 43 key ASVs previously identified via LEfSe analysis and PCo1 differentiation. While PICRUSt2 predicted an overall recovery of microbial sphingolipid metabolism capacity, it failed to pinpoint which core taxa—closely associated with RPT/FPT-induced microbiota structural normalization—functionally underpin this restoration. In addition, serum sphingolipid alterations (reflecting systemic metabolic status) do not directly indicate regulatory effects on brain function. Despite emerging evidence indicating that peripheral sphingolipids can traverse the blood–brain barrier, whereupon they contribute to the activity of amyloid-*β*, it’s not clear whether these circulating metabolites are assumed to directly cross the blood–brain barrier or to indirectly influence brain pathology. Thus, it is essential to further verify the link between peripheral sphingolipid metabolism changes and brain cognitive recovery mechanisms.

**Fig. 5:**
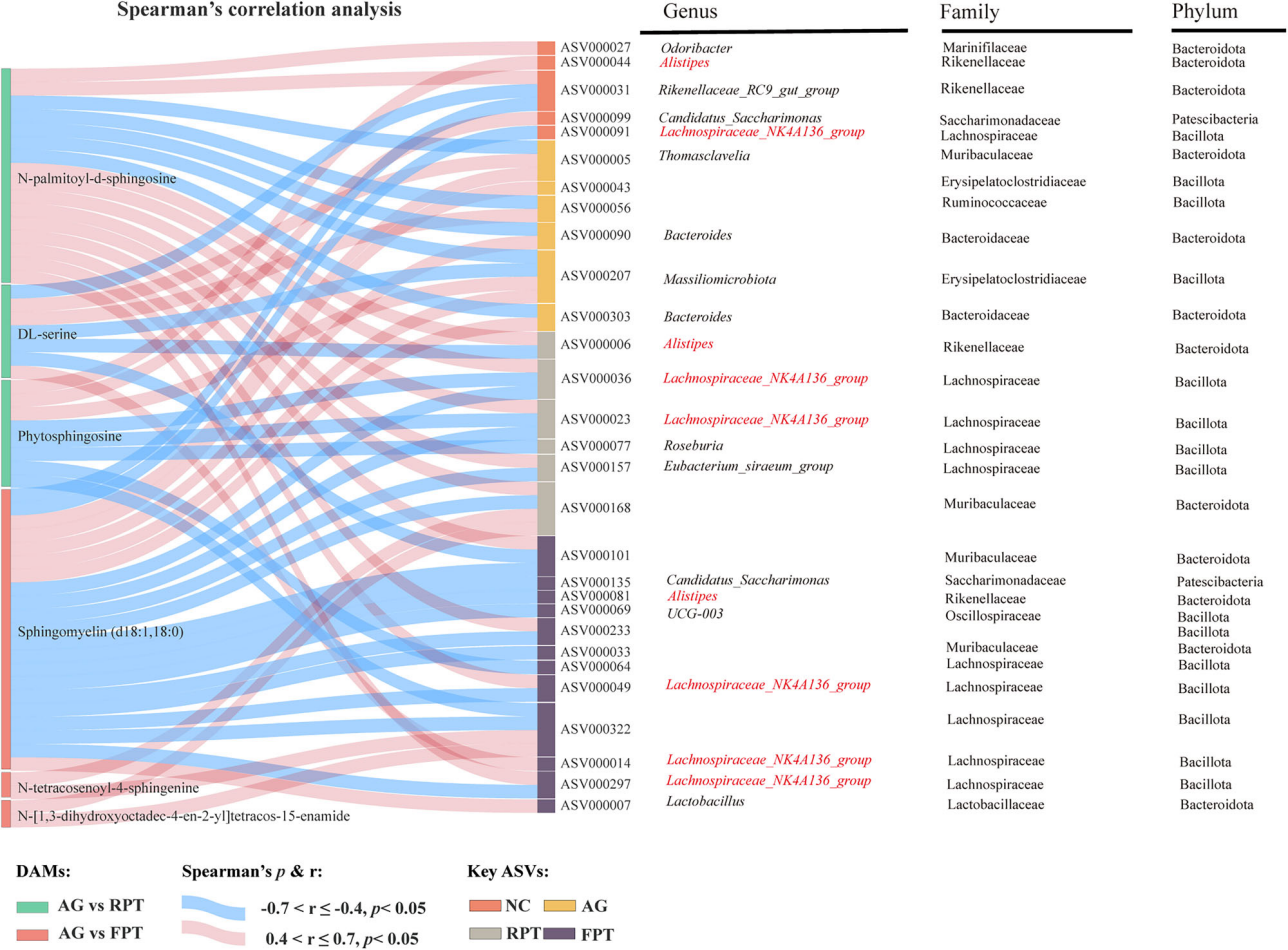
Identifying the key microbial taxa driving sphingolipid metabolism restoration in RPT/FPT-intervened AG mice.

To address the first knowledge gap, we performed correlation analysis between 43 key ASVs and sphingolipid metabolism-related differential metabolites. As depicted in Fig.5, a total of 29 ASVs related to sphingolipid metabolism were identified through the correlations with the differential metabolites from AG vs RPT (N-palmitoyl-d-sphingosine, DL-serine, Phytosphingosine) and AG vs FPT (Sphingomyelin (d18:1,18:0), N-[1,3-dihydroxyoctadec-4-en-2-yl]tetracos-15-enamide, N-tetracosenoyl-4-sphingenine). Notably, three metabolites, N-palmitoyl-d-sphingosine, DL-serine, and Sphingomyelin (d18:1,18:0), were consistently correlated with key ASVs across all four groups. Importantly, their correlation patterns were aligned between the RPT/FPT intervention groups and the NC group, while showing an opposite trend in the AG group. Furthermore, we annotated the taxonomic clusters of these 29 sphingolipid-related ASVs and found that *Lachnospiraceae_NK4A136_group* and *Alistipes* were consistently enriched across the NC, RPT, and FPT groups, which aligned with our earlier observations. In the prior taxonomic profiling, these two taxa were identified as shared biomarkers of microbial homeostasis, being significantly depleted in the AG group but restored to NC-like levels following RPT/FPT intervention. This striking consistency between structural and functional connections strongly suggests that these two core taxa are the key microbial drivers underlying RPT/FPT-mediated sphingolipid metabolism recovery.

**Fig. 6:**
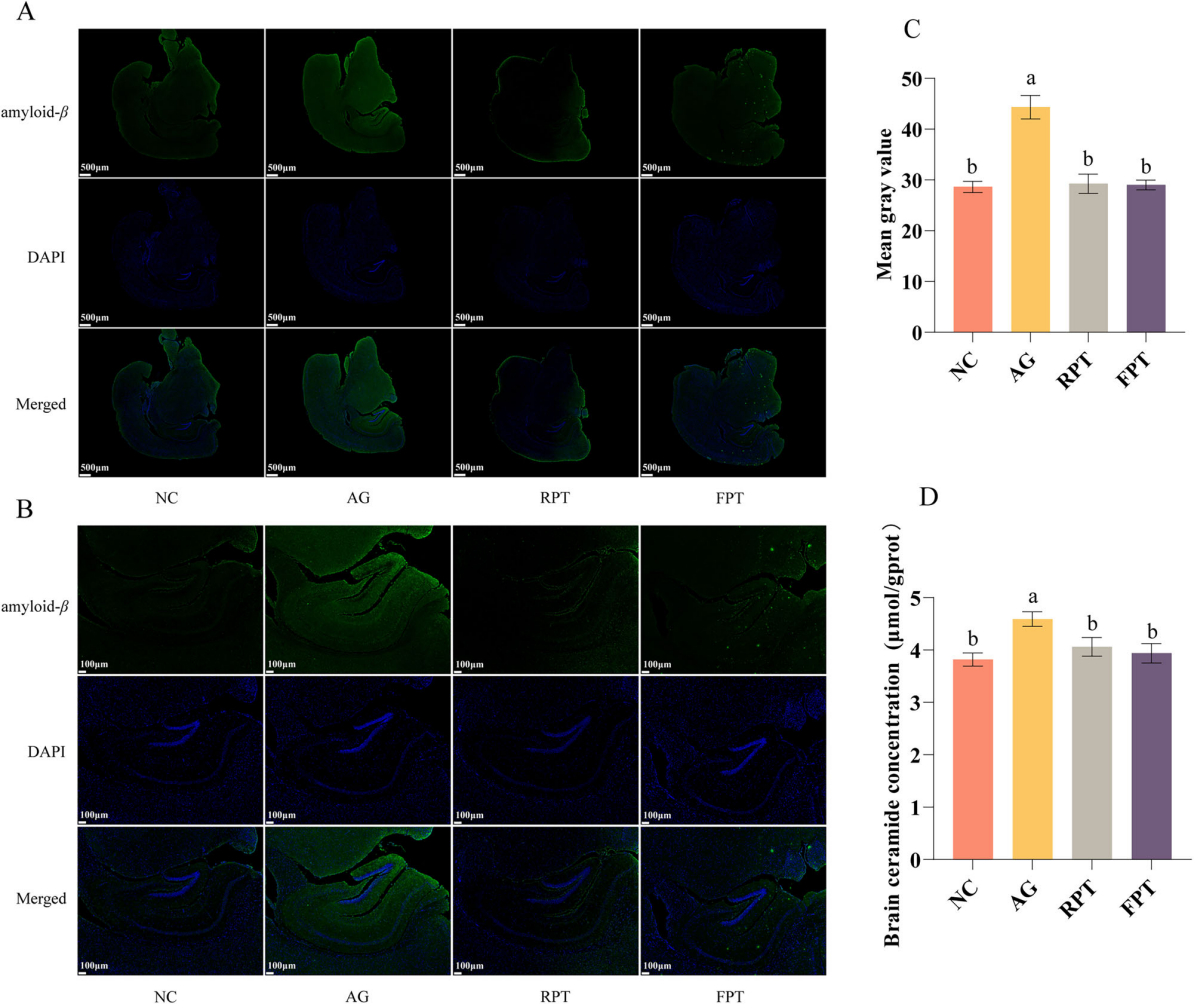
RPT/FPT interventions reduce hippocampal A*β* deposition in aging mice via regulation of sphingolipid metabolism. **A** Representative immunofluorescence images of A*β* deposition (green) in the brain of aging mice; **B** representative immunofluorescence images of A*β* deposition (green) in the hippocampus of aging mice; **C** mean gray value of brain A*β*; **D** brain ceramide concentration.

Previous studies have confirmed disrupted sphingolipid metabolism in Alzheimer’s disease (AD) patients, characterized by elevated serum ceramide levels accompanied by astrocyte activation. Activated astrocytes enhance neuronal susceptibility to A*β*, and both ceramides and A*β* have been demonstrated to participate in binding processes with these cells, collectively driving disease progression^18^. To directly validate whether this mechanism—mediated by peripheral sphingolipid metabolic changes—can influence core pathologies of the central nervous system (addressing the second knowledge gap), we subsequently performed A*β* immunofluorescence staining in the hippocampal region and quantified cerebral ceramide levels in brain tissues. The results indicated that the A*β* immunofluorescence signal was conspicuously enhanced in the brains (especially in the hippocampal region) of AG group mice compared to the NC group, while interventions with both RPT and FPT effectively restored the signal intensity to a level comparable to that of the NC group (Fig. 6A–C). Meanwhile, we observed that the brain ceramide level exhibited a parallel trend to the A*β* immunofluorescence signal. Specifically, the AG group showed a marked elevation in cerebral ceramide content relative to the NC group, whereas both RPT and FPT interventions significantly reduced ceramide accumulation to levels indistinguishable from those in normal controls (Fig. 6D).

Collectively, these results associated the restoration of specific gut microbiota (*Lachnospiraceae_NK4A136_group* and *Alistipes*) with concurrent amelioration of hippocampal sphingolipid-related pathologies (including A*β* deposition and ceramide levels) in AG mice. These coordinated changes suggest a potential link between the gut microbiota and hippocampal sphingolipid metabolism that might underpin the cognitive benefits of RPT and FPT interventions.

### Metabolic profiling and major chemical constituent changes of RPT and FPT infusions

A comparison of the physicochemical compositions between raw Pu-erh tea and ripened Pu-erh tea is pivotal for revealing the link between microbial fermentation and tea bioactivity, and for advancing insights into tea’s functional properties. Therefore, we performed metabolic profiling of RPT and FPT infusions, coupled with quantitative analysis of major chemical constituents, to identify fermentation-mediated changes in their bioactive component profiles.

Firstly, a total of 102 metabolites were identified in the tea extracts via UPLC-Orbitrap-MS analysis, with identification based on comparisons of retention times, mass spectra, and RPT/FPT representative ion chromatograms, plus matching to metabolic databases. Applying the screening threshold (VIP > 1, |log₂FC| ≥ 1, *P* < 0.05)^19^, 28 differential compounds were filtered out, and their details are provided in Table 1. Overall, the pile fermentation triggered profound alterations in the metabolite profile. These 28 differential metabolites spanned multiple classes, including organic acids, phenol acids and their derivatives, amino acids and their derivatives, nucleobases, sugars and sugar derivatives, etc. A core trend of these changes was the marked upregulation of phenol acids, organic acids, and their derivatives, represented by 3,5-Dihydroxy-4-(sulfooxy)benzoic acid, Gallic acid, and D-Tartaric acid. This upregulation might be attributed to microbial enzyme-driven transformations during fermentation^20^, such as the hydrolysis of ester-type catechins to release gallic acid^21^ and the biosynthesis of novel organic acids via microbial central metabolic pathways^22^. Given the well-documented antioxidant, anti-inflammatory, and neuroprotective properties of phenol acids and specific organic acids^23,24^, their accumulation likely enhances the bioactive potential of FPT. Conversely, several metabolites showed significant downregulation, including amino acids (L-Theanine, L-Valine, Pipecolic acid, L-Pyroglutamic acid), central metabolic organic acids (Citric acid, Malic acid), and sugar acids (D-Gluconic acid). This decrease is presumably due to these compounds being utilized as carbon and nitrogen sources by fermenting microorganisms to support their growth and metabolic activities^25^. It is worth noting that several of these downregulated metabolites, such as L-Theanine, are well-recognized neuroprotective constituents of tea^26^, which might exert specific physiological functions linked to cognitive function regulation in RPT.

**Table 1:** 28 differential metabolites of FPT/RPT

No.	Mtabolites	Formula	Mass error [ppm]	*m*/*z*	RT [min]	Adducts	Log_2_FC	VIP	*P* values
1	3,5-Dihydroxy-4-(sulfooxy)benzoic acid	C_5_ H_13_ N O	−3.44	104.10664	0.843	[M + H] + 1	7.32	2.71	4.28E-06
2	Xanthine	C_2_ H_8_ Cl_2_ Si_2_	−3.41	158.9609	0.389	[M + H] + 1	6.73	1.93	8.86E-10
3	D-Tartaric acid	C_6_ H_10_ O S_2_	0.48	323.02749	1.115	[2M−H]−1	5.93	1.85	2.84E-09
4	(2S,4S)-4-hydroxy-2,3,4,5-tetrahydrodipicolinic acid	C_5_ H_4_ O_3_	−2.46	111.00849	1.346	[M−H]−1	4.00	1.66	1.11E-09
5	Choline O-Sulfate	C_7_ H_9_ N O_5_	−3.36	186.04014	1.392	[M−H]−1	3.10	1.16	2.28E-10
6	2-Hydroxyglutaric acid	C_5_ H_4_ N_4_ O_2_	−3.09	151.02568	1.711	[M−H]−1	2.87	1.17	9.75E-09
7	4-Hydroxyprolylthreonine	C_12_ H_22_ O_11_	−4.1	341.10743	0.952	[M−H]−1	2.51	1.21	3.06E-05
8	Uracil	C_9_ H_15_ N_3_ O_2_	−2.85	198.12314	0.802	[M + H] + 1	1.98	1.34	4.94E-10
9	1-Pentofuranosyl-2,4(1H,3H)-pyrimidinedione	C_5_ H_10_ O_6_	−3.11	165.03995	0.831	[M−H]−1	1.67	1.26	1.05E-09
10	Gallic acid	C_7_ H_12_ O_6_	−3.9	191.05535	0.908	[M−H]−1	4.57	5.90	6.96E-06
11	Malic Acid	C_7_ H_6_ O_8_ S	−3.82	248.97011	2.738	[M−H]−1	−1.19	1.21	6.67E-08
12	Citric acid	C_11_ H_20_ O_10_	−3.18	351.06775	0.789	[M + K] + 1	−1.30	2.30	1.11E-09
13	threonic acid	C_10_ H_13_ N_5_ O_4_	−3.13	268.10319	2.63	[M + H] + 1	−1.59	1.23	1.95E-10
14	D-Gluconic acid	C_5_ H_14_ N O_4_ P	−2.9	184.0728	0.813	[M + H] + 1	−1.72	1.40	9.54E-09
15	cyclic 3’,5’-guanosine monophosphate	C_7_ H_15_ N O_3_ S	−3.06	194.08395	0.939	[M + H] + 1	−1.91	1.46	3.18E-10
16	Pipecolic acid	C_6_ H_8_ O_7_	−3.67	191.01902	1.345	[M−H]−1	−2.09	3.19	1.11E-09
17	L-Valine	C_5_ H_7_ N O_3_	−2.87	130.0495	0.818	[M + H] + 1	−2.31	1.54	1.59E-08
18	2-(2-Piperidinyl)ethanesulfonic acid	C_5_ H_8_ O_3_	−3.14	134.0808	0.849	[M + NH4] + 1	−2.34	1.63	1.60E-07
19	L-Proline, 4-hydroxy-5-oxo-4-(tetrahydro-2,3,4-trihydroxy-2-furanyl)-	C_4_ H_8_ O_5_	−2.53	135.02955	0.856	[M−H]−1	−2.98	1.13	4.13E-08
20	Dimethylpyruvic acid	C_4_ H_6_ O_5_	−2.91	133.01386	1.037	[M−H]−1	−3.29	1.99	4.94E-10
21	Sucrose	C_13_ H_16_ O_10_	−3.6	331.06585	2.424	[M−H]−1	−4.18	1.80	3.40E-10
22	L-Theanine	C_4_ H_6_ O_6_	−3.09	149.0087	0.881	[M−H]−1	−4.27	1.17	5.45E-10
23	2-(2-hydroxyethyl)clavam	C_7_ H_11_ N O_3_	−3.79	158.08065	1.228	[M + H] + 1	−4.36	5.78	5.45E-10
24	2-Acetyl-5-pyrroline	C_6_ H_11_ N O_2_	−3.39	130.08582	3.014	[M + H] + 1	−5.15	1.65	8.93E-09
25	L-Pyroglutamic acid	C_5_ H_9_ N O_4_	−2.84	146.0455	0.827	[M−H]−1	−5.66	2.13	3.18E-10
26	Phosphocholine	C_6_ H_9_ N O	−3.47	129.10185	1.228	[M + NH4] + 1	−5.98	1.94	3.18E-10
27	6-O-Galloyl-glucose	C_7_ H_6_ O_5_	−3.73	169.0136	2.763	[M−H]−1	−6.58	4.04	1.38E-10
28	1,4,8,9,10,11,12,13-Octahydroxy-3-(hydroxymethyl)-3,4,4a,16a-tetrahydro-1H-dibenzo[f,h]pyrano[3,4-b][1,4]dioxecine-6,15-dione	C_5_ H_11_ N O_2_	−3.02	118.0859	1.143	[M + H] + 1	−7.11	1.19	4.94E-10

*P* values are presented in scientific notation following false discovery rate (FDR) adjustment for multiple comparisons. All listed metabolites are statistically significant (adjusted *p* < 0.05).

Secondly, quantitative analysis was performed on the bioactive substances of tea. As presented in Table 2, pile fermentation profoundly reshapes the functional component profiles of RPT and FPT. The core differences and underlying mechanisms are elaborated as follows. In terms of active components, multiple key substances in FPT exhibit significant reductions: free amino acids, tea polyphenols, and L-theanine all decrease to varying degrees. Among catechins, EGC, EGCG, GCG, and ECG are drastically depleted to levels below the instrument detection limit (nearly undetectable); EC remains detectable but with a marked content reduction, resulting in a substantial decline in total catechins. Regarding tea pigments, theaflavin content shows no significant fluctuation compared with RPT, while thearubigin content decreases remarkably, and theabrownin content increases sharply. Concurrently, the contents of theobromine, caffeine, and gallic acid in FPT display distinct upward trends. The variation trends of partial components are consistent with the aforementioned UPLC-Orbitrap-MS analysis results, verifying the reliability of the data. Collectively, these changes in functional components provide a basis for further exploring the pharmacological effects related to RPT and FPT.

**Table 2:** Main bioactive constituents of RPT and FPT

Constituent	RPT infusion (mg/L)	FPT infusion (mg/L)
Free amino acid	380.57 ± 3.38	186.17 ± 3.76*
Tea polyphenols	1439.16 ± 10.16	851.08 ± 8.89*
Theaflavins	10.37 ± 0.68	9.10 ± 0.34
Thearubigins	473.48 ± 9.75	68.91 ± 7.45*
Theabrownins	191.60 ± 3.68	1035.49 ± 7.45*
L-Theanine	181.10 ± 3.65	35.10 ± 0.97*
Theophylline	–	–
Theobromine	32.00 ± 0.45	35.57 ± 0.96
Caffeine	467.51 ± 3.83	556.32 ± 10.88*
Gallic acid	29.47 ± 0.27	130.39 ± 2.60*
EGC	146.45 ± 12.53	–*
EC	114.30 ± 0.85	14.84 ± 0.98*
EGCG	483.82 ± 4.40	–*
GCG	56.67 ± 7.16	–*
ECG	329.11 ± 4.76	–*
Total catechins	1130.39 ± 19.11	14.84 ± 0.98*

– means that the substance is below the detection limit. Data were assessed by *t*-test, values with letter “*” representing statistically significant results compared with the RPT group (*P* < 0.05).

### Identifying core cognitive-improving components in RPT/FPT

To clarify the key bioactive components underlying the cognitive-improving effects of RPT and FPT in D-galactose-induced AG mice, we conducted a targeted correlation analysis between tea chemical constituents and cognitive function/mechanistic indicators. A critical limitation of relying solely on RPT and FPT is that constituent changes driven by fermentation may accidentally align with trends in physiological indicators (trend-based spurious correlations), rather than reflecting true functional relevance, which could lead to misidentifying non-core components.

**Fig. 7:**
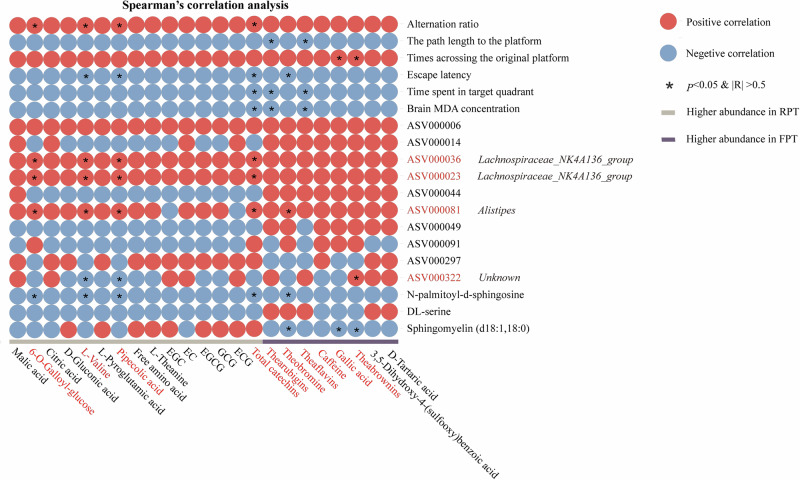
Identifying core cognitive-improving components in RPT/FPT by correlating tea chemical constituents and cognitive function / mechanistic indicators.

To address this limitation and boost the reliability of core component screening, we incorporated data from two additional aged raw Pu-erh tea samples (RPT-2004 and RPT-2014, which, together with RPT, were processed and manufactured by the same company) processed in the same animal experimental batch. Our goal was not to evaluate the cognitive effects of tea aging itself. Instead, we used these aged samples as an independent validation cohort to strengthen our correlation analysis. By doing so, we effectively filtered out non-specific, trend-driven associations, allowing us to confidently identify and confirm the components truly linked to the cognitive benefits of RPT and FPT (the effective intake doses of these key components in the mouse experiment are detailed in Table 3). As shown in Fig. 7. Through Spearman’s correlation analysis, we found that several tea chemical constituents exhibited notable correlations with cognitive function and mechanistic indicators. For RPT, components like 6-O-Galloyl-glucose, L-Valine, Pipecolic acid, and total catechins showed significant correlations with cognition-related indicators, strongly suggesting their crucial roles in improving cognitive performance. Specifically, these RPT-related functional components were correlated with key ASVs, which were classified within the genus *Lachnospiraceae_NK4A136_group* and *Alistipes* — both of which are the key microbial taxa we previously identified as being involved in cognitive impairment improvement. In contrast, for FPT, compounds including thearubigins, theobromine, theaflavins, gallic acid, and theabrownins were significantly correlated with relevant cognitive and mechanistic markers, indicating their potential as functional components underlying its cognitive-improving effects. These FPT-associated functional compounds were also found to correlate with ASVs that fell into *Alistipes*, another key microbial genus we identified for alleviating cognitive impairment. Collectively, these findings highlight that specific tea components from RPT and FPT, by interacting with these previously confirmed key microbial taxa, may exert prominent effects on cognitive improvement.

**Table 3:** Effective doses of the main neuroprotective compounds

Constituent	Mouse (mg/kg/day)	
	RPT	FPT
Tea polyphenols	113.6	67.2
Theaflavins	0.82	0.72
Thearubigins	37.4	5.5
Theabrownins	15.1	81.8
Theobromine	2.53	2.81
Caffeine	36.9	43.9
Gallic acid	2.33	10.29
Total catechins	89.24	1.17

Doses were calculated based on an average daily intake of 3.0 mL of tea infusion per mouse and an average body weight of 38.0 g, which are the mean values from mice treated with RPT and FPT.

It should be noted that total catechins—comprising individual monomers (EGC, EC, EGCG, GCG, and ECG)—showed a significant positive correlation with cognitive markers, whereas none of these individual catechins did so independently. However, this finding does not mean the individual monomers are inactive. Two plausible mechanisms might account for this discrepancy. Firstly, a bioactive concentration threshold effect is likely involved. Each catechin monomer in RPT exists at a concentration too low to reach the threshold required for a detectable correlation with cognitive markers^27^. However, their cumulative content as total catechins surpasses this threshold, thereby enabling a significant association with cognitive improvement. Secondly, coordinative or synergistic interactions between the monomers may drive the outcome^28,29^. Each catechin may exert a weak but real regulatory effect on cognitive-related pathways. When acting together, these weak individual effects are amplified to a magnitude that becomes statistically significant in correlation with cognitive markers.

In sum, RPT and FPT rely on distinct sets of core components to exert their cognitive-improving effects: RPT depends on 6-O-Galloyl-glucose, L-Valine, Pipecolic acid, and total catechins (with the latter’s efficacy driven by either concentration threshold or monomer synergy), while FPT relies on tea pigments (thearubigins, theaflavins, theabrownins), alkaloids (theobromine), and gallic acid. This finding not only clarifies the bioactive basis of RPT/FPT’s cognitive benefits but also highlights the importance of considering both individual components and their collective effects in natural product research.

## Discussion

In the present study, we assessed the effects of ad libitum access to RPT and FPT infusions—an intervention that simulates habitual human tea consumption—on the amelioration of cognitive impairment in D-galactose-induced aging mice. Our analyses yielded four principal findings.

To begin with, both RPT and FPT could significantly improve cognitive functions in aging mice, with fermentation status showing no notable impact on the cognitive-enhancing effects. Subcutaneous injection of D-galactose is a well-established method for constructing aging models characterized by oxidative stress and memory impairment^30,31^. In the current study, 8 weeks of subcutaneous D-galactose injection induced memory dysfunction, hippocampal histopathological alterations, and brain oxidative stress in mice. These adverse alterations were rescued by RPT and FPT intervention, with comparable ameliorative efficacy between the two groups. Specifically, both groups exhibited: (1) a similar increase in spontaneous alternation rate in the Y-maze test; (2) comparably reduced total swimming path length and escape latency, alongside increased platform crossings and time spent in the target quadrant in the Morris water maze test; (3) a similar elevation in hippocampal neuron count and restoration of dentate gyrus morphology; and (4) a comparable decrease in cerebral MDA content. Notably, a significant increase in cerebral GSH levels was exclusively observed in the FPT intervention group, suggesting a specific regulatory mechanism of FPT in modulating cerebral antioxidant capacity.

Additionally, RPT and FPT could reverse D-galactose-induced intestinal flora disturbance. Research has confirmed that the gut microbiome is intimately linked to host health, owing to its central role in metabolism and immune regulation. From childhood to adulthood, gut microbial diversity gradually establishes and stabilizes. However, during aging, the gut microbiome undergoes marked alterations in composition and metabolic activity, characterized by increased abundance of subdominant species and rearrangement of microbial co-occurrence networks^32^. In D-galactose-induced aging models, a decrease in relative abundance and diversity of the intestinal flora can be observed^33,34^. Consistent with these findings, we also observed the same phenomenon in our study. Specifically, such modeling treatment induced a decline of the Shannon index and PD-tree index of intestinal microbiota, indicating an obvious reduction of gut microbial diversity. Meanwhile, UPGMA clustering and PCoA results further revealed that the overall structure of the intestinal microbial community in AG mice was distinctly separated from that in NC mice, reflecting a pathological shift accompanied by cognitive impairment. Fortunately, such a pathological shift was restored by RPT and FPT, with the microbial profile approaching that of NC mice. Furthermore, we identified *Lachnospiraceae_NK4A136_group*, *Alistipes* as the core taxa that were consistently enriched in both RPT and FPT intervention groups, as well as in the NC group, while being significantly depleted in AG mice. This observation is consistent with previous studies, which have demonstrated that the abundances of *Lachnospiraceae* and *Alistipes* tend to decrease in aging-associated gut microbial dysbiosis^35–39^. Notably, dietary polyphenols have been demonstrated to positively modulate the abundance of these bacterial groups^12,40^.

Mechanistically, the alleviation of cognitive impairment by RPT and FPT may be associated with the modulation of the “gut microbiota–sphingolipid metabolism–brain” axis. Our serum metabolomic analysis revealed a significant disturbance in sphingolipid metabolism in the D-galactose-induced aging model mice, which was notably reversed following RPT and FPT intervention. Dysregulation of sphingolipid metabolism, particularly that of ceramides, is recognized as a key pathological feature of AD^18,41^. It is noteworthy that circulating ceramides, owing to their lipophilic nature, are thought to traverse the blood–brain barrier and accumulate in the central nervous system, potentially leading directly to neuronal damage^42^. Concurrently, abnormal elevation of endogenous ceramides in the brain can promote the generation of A*β* by enhancing the stability of *β*-secretase 1, thereby driving core AD pathology^43^. In this study, we identified the differential serum metabolites N-palmitoyl-D-sphingosine between the aging model group and the RPT group, as well as N-[1,3-dihydroxyoctadec-4-en-2-yl]tetracos-15-enamide and N-tetracosenoyl-4-sphingenine between the aging model group and the FPT group. These were all characterized as ceramides, and their serum levels were significantly reduced after the respective tea interventions. Further analysis of brain tissue confirmed that the concentration of ceramides in the brain was synchronously decreased following RPT and FPT intervention. In parallel, immunofluorescence staining of A*β* in the hippocampal region showed a marked reduction in signal intensity post-intervention. Besides, integration with gut microbiota data further indicated that the restoration of sphingolipid metabolism was functionally linked to key microbial taxa, particularly *Lachnospiraceae_NK4A136_group* and *Alistipes*. These observations collectively suggest that tea interventions may exert comprehensive benefits via the “gut microbiota–sphingolipid metabolism–brain” axis. Nevertheless, the direction of causality among these interconnected variables requires further elucidation through targeted experimental designs.

Last but not least, RPT and FPT exerted these neuroprotective effects via polyphenols and their derivatives. Pile fermentation, a crucial solid-state microbial process in dark tea production, profoundly reshapes the functional chemical profile of tea leaves through enzymatic catalysis driven by specific microorganisms^44^. In our present study, tea metabolic comparison confirmed that pile fermentation led to a significant reduction in tea polyphenols and catechins, while promoting the accumulation of fermentation-derived compounds such as tea pigments and gallic acid, which aligned with prior findings^45,46^. These compositional shifts were functionally linked to cognitive improvement through correlation analysis, which identified FPT-specific core components—including thearubigins, theaflavins, theabrownins, theobromine, and gallic acid—as key contributors to the amelioration of cognitive impairment in aging mice. Conversely, RPT’s effects were associated with distinct components such as 6-O-Galloyl-glucose, L-Valine, and total catechins. Notably, although pile fermentation mediated a reconstruction of the bioactive constituents in RPT and FPT, an undeniable fact is that their core cognitive-improving components are intrinsically connected with polyphenols and their derivatives. For example, in RPT, the polyphenolic profile is predominantly comprised of unoxidized, monomeric catechins, such as EGC, EC, EGCG, GCG, and ECG. In contrast, the microbial enzymatic-driven transformation during pile-fermentation converts these monomers into dimeric and oligomeric polyphenolic compounds in FPT, including thearubigins, theaflavins, and theabrownins, alongside polyphenol metabolites like gallic acid. Among the components, many monomers^4^, including EGCG^47^, ECG^3^, theaflavins^13^, theabrownins^48^, have been proven to prevent and delay aging by modulating gut microbiota. These compositional differences are expected to result in distinct intestinal absorption and microbial metabolism. However, their comparable neuroprotective efficacy suggests a potential metabolic convergence driven by gut microbiota. Specifically, despite their distinct initial structures, both classes of compounds are polyphenols and their derivatives. Emerging evidence indicates they undergo convergent microbial transformations in the gastrointestinal tract, potentially yielding a shared spectrum of bioactive metabolites responsible for the final biological effects^49^. A key example is the hydrolysis of ester-type catechins: during pile fermentation, microbial esterases pre-digest compounds like EGCG into EGC and gallic acid—a reaction that also occurs in vivo upon ingestion of RPT. Thus, pile fermentation can be viewed as an external pre-digestion process that accelerates an intrinsic metabolic step. More broadly, both monomeric catechins in RPT and polymeric pigments in FPT serve as substrates for colonic microbiota. A growing consensus suggests that diverse dietary polyphenols are funneled by gut microbes into a limited, overlapping pool of small phenolic acid metabolites (e.g., phenyl-*γ*-valerolactones)^50,51^. Consequently, despite different starting points, both teas may ultimately generate similar profiles of active metabolites through microbiota-dependent transformation. These shared bioactive metabolites likely contribute to the observed cognitive benefits by modulating key microbial taxa, particularly *Lachnospiraceae_NK4A136_group* and *Alistipes*^12,40^, which were consistently restored in both intervention groups. The subsequent improvement in gut-brain communication may thereby ameliorate cognitive impairment in aging mice. Therefore, we conclude that despite the marked differences in bioactive constituents between RPT and FPT induced by pile fermentation, the core cognitive-improving effects of both RPT and FPT are mainly driven by polyphenols and their derivatives.

To summarize, our investigation outcomes demonstrated that ad libitum access to RPT and FPT could exert profound cognitive-improving effects in D-galactose-induced aging mice. The key finding is that their neuroprotective effects are mediated through the modulation of the “gut microbiota–sphingolipid metabolism–brain” axis. Surprisingly, although RPT and FPT showed distinctive differences in terms of bioactive ingredients, they exerted comparable properties in attenuating cognitive impairment, reversing gut microbiota disturbance, and restoring sphingolipid metabolism homeostasis. The underlying mechanisms could be that RPT and FT displayed similar effects in promoting the proliferation of core beneficial taxa (specifically, *Lachnospiraceae_NK4A136_group* and *Alistipes*) and in downregulating pathogenic ceramide levels. Furthermore, tea chemical constituent analyses exhibited that microbial fermentation caused a fundamental reconstruction of the polyphenol profile: RPT’s benefits were linked to its native monomeric catechins, while FPT’s effects were driven by fermentation-derived polymeric pigments and gallic acid. However, this difference in polyphenol forms did not remarkably influence their core efficacy in alleviating cognitive impairment, as both categories of polyphenolic compounds are intrinsically connected to the regulation of the gut-brain axis. Together, the current data supply valuable information on the relationship among the microbial fermentation of tea, tea bioactivities, and cognitive health, underscoring that both raw Pu-erh tea and ripened Pu-erh teas can serve as effective dietary interventions for mitigating age-related cognitive decline via polyphenol-driven gut-brain communication.

## Methods

### Brain assays

D-galactose was purchased from Sigma-Aldrich (St. Louis, MO, USA). D-Malondialdehyde (MDA), glutathione (GSH), and superoxide dismutase (SOD) commercial kits were obtained from Nanjing Jiancheng Bioengineering Institute (Nanjing, China), and the mouse ceramide ELISA kit was from Shanghai Enzyme-linked Biotechnology Co., Ltd. (Shanghai, China). All kits are measured in strict accordance with the instructions.

### Experimental materials

The fresh tea leaves (*Camellia sinensis* var. *assamica*) were sourced from Xiaguan, Yunnan, China, and both raw Pu-erh tea (RPT) and ripened Pu-erh tea (FPT) were produced using sun-dried raw tea (SRT) as the base material, with manufacturing procedures referring to the Chinese national standard (GB/T 9833.5-2013) with slight modifications: the fresh leaves were first processed via spreading, enzyme inactivation, rolling, and ball-breaking, followed by sun-drying to produce the base SRT; for RPT, the SRT was directly steamed, compressed and dried into the final product, whereas for FPT, the SRT was subjected to pile fermentation (moistened with 30–40% water), after which the fermented tea was steamed and compressed then dried to obtain the final FPT product.

### Preparation of tea infusion

Based on previous analysis of tea concentration in daily scenarios, tea infusion concentration was selected for the intervention. The tea infusion was prepared with slight modifications according to the sensory evaluation method for tea (GB/T 23776—2018). Specifically, tea leaves were placed in a beaker and steeped in boiling water for 5 miutes at a ratio of tea to distilled water of 1 g: 50 ml. The tea infusion was collected after two rounds of filtration and stored at −80 °C until subsequent experiments. Before use, the tea infusion was thawed to ensure stability.

### Animals and experimental design

Thirty-two 8-week-old male specific-pathogen-free Institute of Cancer Research mice were purchased from SJA Laboratory Animal Co., Ltd. (Hunan, China) for this study. All mice were housed in a standardized environment maintained at 23 ± 2 °C with 50 ± 10% relative humidity and a 12 h light/dark cycle, and were given free access to an identical standard maintenance diet (SY10001F, Shuyu Biotechnology Co., Ltd., Shanghai, China) and drinking water throughout the experiment.

Following a 7-day acclimation period to minimize stress, the mice were stratified by body weight and then randomly assigned to four groups (*n* = 8 per group): normal control (NC), aged control (AG), RPT intervention (RPT), and FPT intervention (FPT). The NC group received a daily subcutaneous injection of 0.3 mL 0.9% physiological saline and had free access to distilled water. In contrast, the AG, RPT, and FPT groups were subcutaneously injected with D-galactose at a dose of 150 mg/kg body weight daily to induce an aging model. Among these three groups, the AG group was provided with distilled water, while the RPT and FPT groups were given RPT infusion and FPT infusion, respectively, as their exclusive drinking fluids (ad libitum).

During the 8-week experimental period, daily recordings were made of the mice’s voluntary intake of water or tea infusions, and weekly measurements were taken of their body weight and food intake. After 7 weeks of intervention, the Y-maze and Morris water maze test were conducted to evaluate spatial learning and memory. At the end of the 8th week, all mice were fasted overnight, anesthetized via intraperitoneal injection of pentobarbital sodium (50 mg/kg), and euthanized by cervical dislocation. Brain tissue, cecal contents, and serum samples were immediately collected from each mouse and stored at −80 °C for subsequent laboratory analyses (Fig. 1A). Data acquisition and subsequent analysis were conducted in a single-blind manner.

All animal experiments were conducted in strict accordance with the Chinese Laboratory Animal Management regulations, adhering to the guidelines for the Care and Use of Laboratory Animals, and with the approval granted by the Laboratory Animal Welfare and Ethics Committee of Hunan Agricultural University (Certificate No. 2021-2137: Changsha, Hunan, China).

### Hematoxylin and eosin staining of the hippocampus

Hippocampal tissues were first fixed in 4% paraformaldehyde infusion for 24 h to preserve their structural integrity. After fixation, the tissues underwent a standard histological processing workflow: sequential dehydration through graded ethanol infusions, clearing in xylene, and embedding in paraffin wax to form solid tissue blocks. These paraffin blocks were then sectioned into thin slices (3–4 μm thick) using a microtome to ensure optimal visualization of cellular details. The resulting paraffin sections were deparaffinized and rehydrated before being stained with H&E. Stained sections were finally observed and imaged under a light microscope (Nikon Eclipse Ci, Nikon, Tokyo, Japan) equipped with a digital camera, allowing for detailed examination of hippocampal tissue morphology.

### Immunofluorescence staining

Mouse brain paraffin sections were dewaxed, rehydrated, and subjected to antigen retrieval. In brief, after heat-induced antigen retrieval using citrate antigen retrieval buffer (pH 6.0), the sections were blocked with 10% goat serum (PN0038, Pinuofei Bio) at 37 °C for 30 min. Subsequently, the sections were incubated overnight at 4 °C with the primary antibody diluted in antibody diluent (PN1023, Pinuofei Bio). The primary antibody used in this study was rabbit anti-*β*-amyloid (A*β*) (GB111197-100, Saiveier Bio, 1:100 dilution). The following day, after washing with PBS (PN0031, Pinuofei Bio), the sections were incubated with Alexa Fluor 488-conjugated goat anti-rabbit secondary antibody (PN0108, Pinuofei Bio, diluted according to the manufacturer’s instructions) at 37 °C for 1 h in the dark. Finally, cell nuclei were counterstained with DAPI (PN0015, Pinuofei Bio), and the sections were mounted with an anti-fade mounting medium (PN0024, Pinuofei Bio). All fluorescence images were captured using an inverted fluorescence microscope (Nikon, Tokyo, Japan). The fluorescence intensity of A*β* was quantified using Image-Pro Plus 6.0 software (Media Cybernetics, MD, USA).

### Y-maze spontaneous alternation test

The Y-maze spontaneous alternation test was employed to assess short-term spatial memory in mice, capitalizing on the inherent propensity of rodents to prioritize exploration of novel arms over those they had previously visited. The Y-maze apparatus comprises three identical arms intersecting at a 120° angle at the central hub. For the experimental procedure, each mouse was first placed at the center of the three-arm intersection and permitted 5 min of free exploration to acclimate to the maze environment. Following a 1-h interval, the formal test was initiated: the mouse was repositioned at the central intersection and allowed to explore the maze freely for 8 min. The spontaneous alternation ratio was quantified using the formula: The spontaneous alternation rate = Alternation number/(total number of arms entered − 2), where “alternations” refer to consecutive entries into three distinct arms during the test period.

### Morris water maze test

The Morris water maze test was conducted to evaluate spatial learning and memory in mice, based on the principle that rodents avoid water and will actively learn to locate a hidden escape platform using spatial cues placed around the maze. The apparatus consisted of a circular pool, photosensors, and a computerized video-tracking module. One day before the formal test, all mice underwent acclimation: they were placed on a visible platform in an undyed pool and allowed to stay for 60 s to adapt to the water environment and recognize the platform. The formal test, conducted following the protocol described by published paper^13^, included two phases: the hidden platform phase (Day 6) and the probe test phase (Day 7). For the hidden platform phase, the pool water was dyed white to make the platform invisible; mice were randomly placed into the pool to search for the platform, with parameters including escape latency, average swimming speed, time spent in the target quadrant, and number of crossings over the original platform recorded. For the probe test phase, the platform was removed, and each mouse was allowed to search the pool freely for 60 s, with the same parameters (number of crossings over the former platform area, average swimming speed, time spent in the target quadrant) recorded to evaluate consolidated spatial memory.

### 16S rRNA gene sequencing and bioinformatics analysis

The 16S rRNA gene sequencing for mice cecal contents was performed by Guangzhou Gene Denovo Biotechnology Co., LTD., with detailed procedures referred to the published study^11^.

### Nontargeted metabolomics analysis of serum

Serum nontargeted metabolomics analysis was conducted by Guangzhou Gene Denovo Biotechnology Co., LTD., with detailed procedures referred to the published study^52^.

### Determination of the chemical components of tea infusion

To explore the regulatory effects of pile fermentation—the key differential process between raw and ripened Pu-erh tea—on the metabolic profile of tea infusions, we conducted quantitative analysis on the main functional components with neuroprotective effects in tea. Specifically, the contents of free amino acids and tea polyphenols were quantified according to the method described by Zhou et al.^53^; for specific components—including theabrownins, theobromine, caffeine, epigallocatechin (EGC), epicatechin (EC), epigallocatechin gallate (EGCG), (−)-gallocatechin (GCG), and epicatechin gallate (ECG)—their quantitative determination followed the protocol reported by Gong et al.^54^; Additionally, the contents of theabrownins, theaflavins, and thearubigins were quantified using the method established by Wang et al.^55^. To further clarify how fermentation drives metabolic variations, an ultra-high-performance liquid chromatography-Orbitrap-mass spectrometry (UPLC-Orbitrap-MS) system was utilized for qualitative and semi-quantitative analysis of chemical components, with the entire experimental operation carried out in strict accordance with the technical protocol described in the published study^52^.

### Statistical analysis

In the present study, one-way analysis of variance along with Fisher’s LSD test was used to undertake the statistical analysis of the column chart data with the aid of GraphPad Prism 9. Data were presented as the mean ± standard error of the mean ($\bar{x}\,$± SEM), and values with different letters represent statistically significant results. Online platforms OmicShare, Omicsmart (https://www.omicshare.com/tools, https://www.omicshare.com) were used to analyze the 16 rRNA gene sequencing and serum nontargeted metabolomics data. The correlations were analyzed using Spearman’s correlation method through IBM SPSS Statistics 25, and *P* < 0.05 was considered significant.

## Supplementary information

41538_2026_872_MOESM1_ESM

## Data Availability

The datasets produced from the study are available from the corresponding authors on reasonable request.
