# Supplementary material for: Comparable neuroprotection efficacy of raw Pu-erh tea and ripened Pu-erh tea in D-galactose-induced aging mice via gut-brain axis
Source: NPJ Sci Food. 2026 May 5;10:218. doi: 10.1038/s41538-026-00872-x (PMC13350881; doi:10.1038/s41538-026-00872-x)
Supplement: Supplementary file 1 — 41538_2026_872_MOESM1_ESM [file 41538_2026_872_MOESM1_ESM.docx]

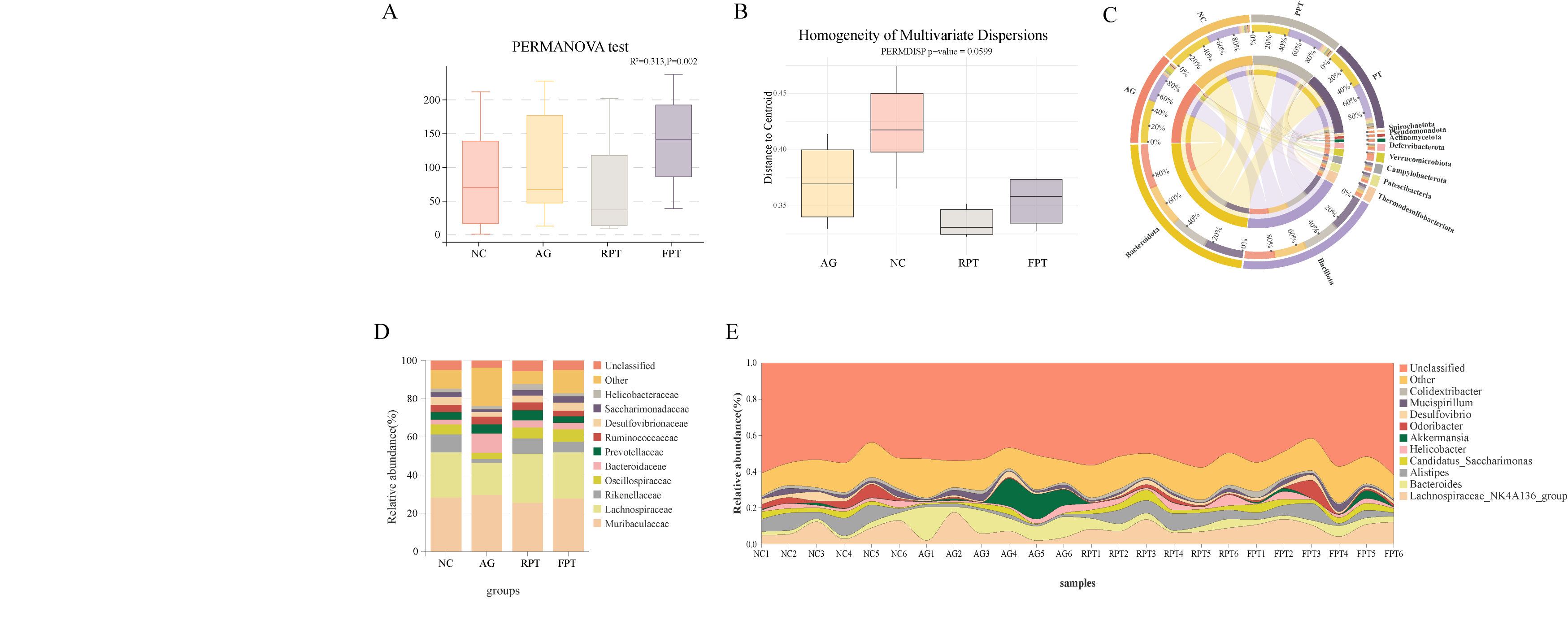


Fig.S1 PERMANOVA, PERMDISP results and gut microbial profiles at the phylum, family, and genus taxonomic levels. (A) PERMANOVA based on Bray-Curtis distances testing the overall significance of community structure differences among groups. (B) PERMDISP testing the homogeneity of multivariate dispersions across groups; (C) gut microbial profiles at the phylum level; (D) gut microbial profiles at the family level; (E) gut microbial profiles at the genus level.

Fig.S2 LEfSe analysis on identified 47 ASVs as differentially abundant across the NC, AG, RPT, and FPT groups

­­­
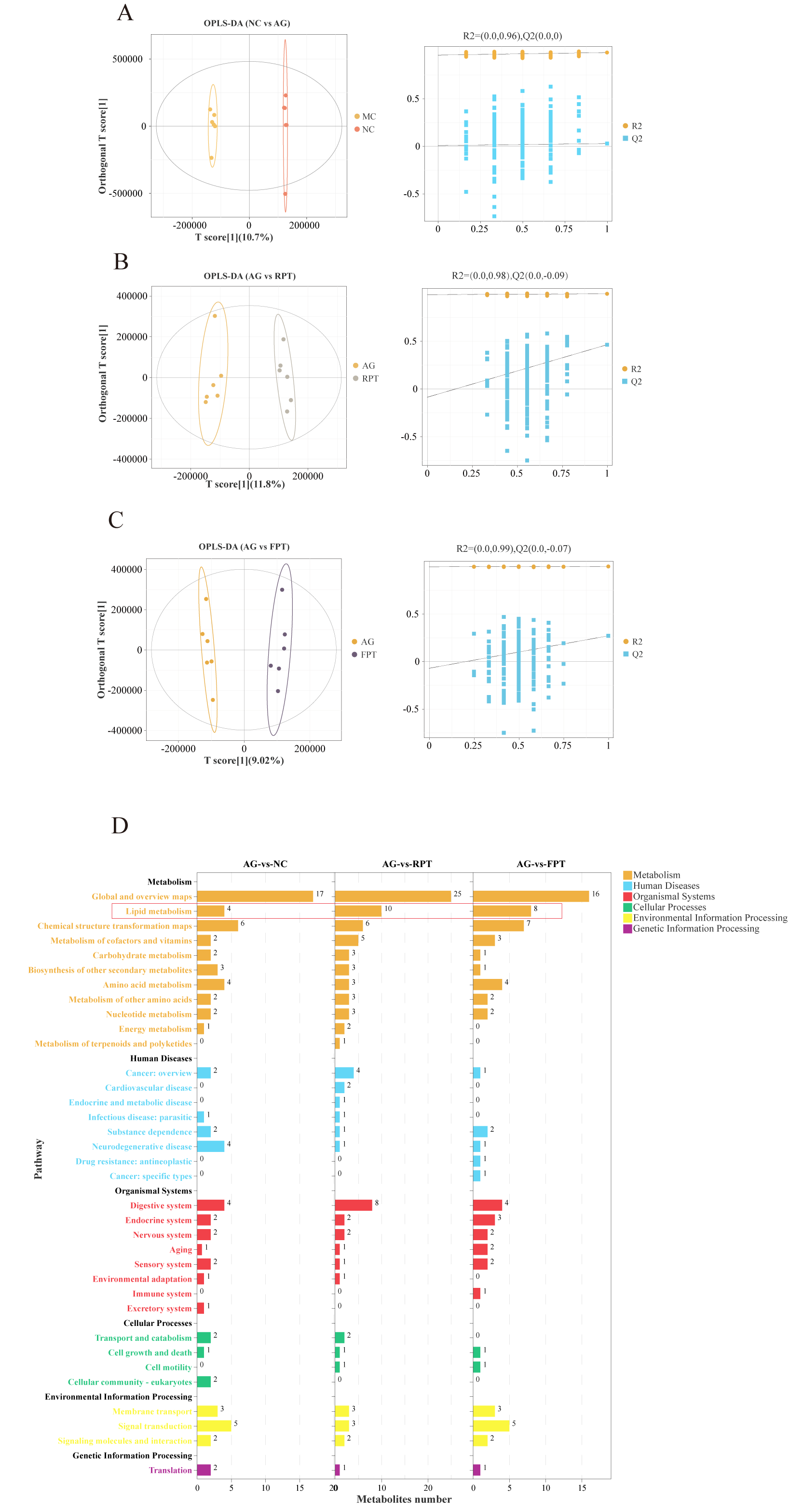


Fig.S3 OPLS-DA analysis and KEGG pathway classification of serum metabolites. (A) OPLS-DA and permutation test results of NC vs AG. (B) OPLS-DA and permutation test results of AG vs RPT; (C) OPLS-DA and permutation test results of AG vs FPT; (D) KEGG pathway classification of serum metabolites.


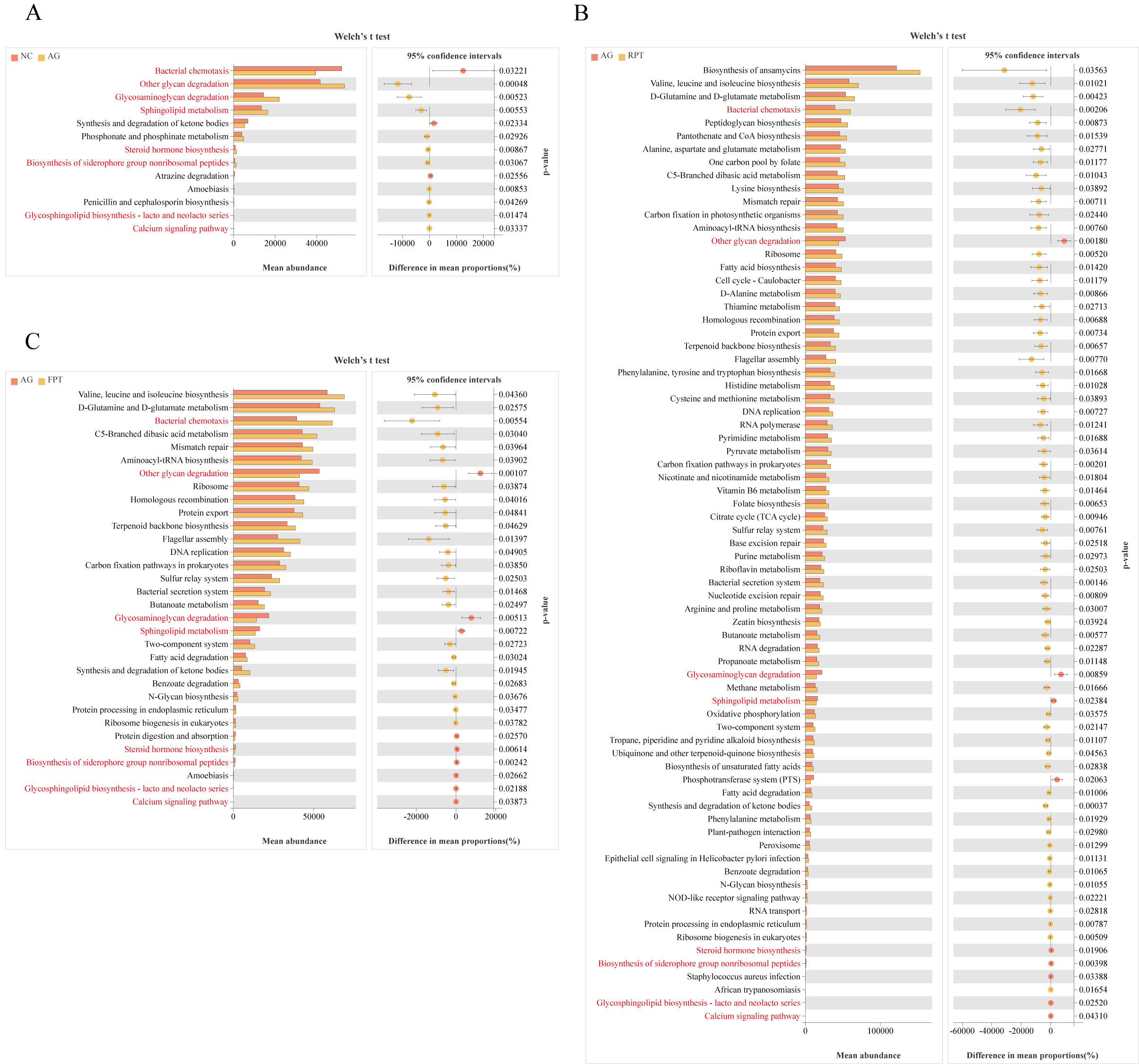


Fig.S4 Predictive profiling of gut microbiota functional potential via PICRUSt2. (A) PICRUSt2 results of NC vs AG. (B) PICRUSt2 results of AG vs RPT; (C) PICRUSt2 results of AG vs FPT

**Supplementary table. 1.** **61 vital endogenous metabolites affected by RPT intervention**

| **NO.** | **Metabolites** | **Class** | **RPT/MC** | | |
| --- | --- | --- | --- | --- | --- |
|  |  |  | **Log_2_FC** | **VIP** | ***p*-value** |
| 1 | Ps 34:2 | Glycerophosphoserines | 0.8 | 1.36 | 0.03 |
| 2 | Pi 34:2 | Glycerophosphoinositols | -0.63 | 4.53 | 0.01 |
| 3 | Pi 36:4 | Glycerophosphoinositols | -0.32 | 5.28 | 0.01 |
| 4 | Pi(17:0,18-hete) | Glycerophosphoinositols | 0.78 | 2.67 | 0.01 |
| 5 | Pg 38:4 | Glycerophosphoglycerols | 1.09 | 1.22 | 0.05 |
| 6 | Pg 38:6 | Glycerophosphoglycerols | 0.91 | 1.59 | 0 |
| 7 | Pg 36:3 | Glycerophosphoglycerols | 0.41 | 1.08 | 0.05 |
| 8 | Pg 40:8 | Glycerophosphoglycerols | 0.89 | 1.48 | 0.03 |
| 9 | 1-palmitoyl-2-oleoyl-sn-glycero-3-phosphoethanolamine | Glycerophosphoethanolamines | 0.7 | 2.26 | 0.05 |
| 10 | 2-docosahexaenoyl-1-palmitoyl-sn-glycero-3-phosphoethanolamine | Glycerophosphoethanolamines | 1.25 | 2.64 | 0.03 |
| 11 | 2-arachidonoyl-1-stearoyl-sn-glycero-3-phosphoethanolamine | Glycerophosphoethanolamines | 0.88 | 12.18 | 0.01 |
| 12 | Pe 36:2 | Glycerophosphoethanolamines | 0.67 | 9.02 | 0.05 |
| 13 | Pe 36:4 | Glycerophosphoethanolamines | 0.83 | 4.97 | 0.04 |
| 14 | Pe(16:1e,14-hdohe) | Glycerophosphoethanolamines | 0.9 | 1.85 | 0.02 |
| 15 | (2-aminoethoxy)[2-[hexadec-9-enoyloxy]-3-[octadeca-1.11-dien-1-yloxy]propoxy]phosphinic acid | Glycerophosphoethanolamines | -0.42 | 2.09 | 0.02 |
| 16 | (2-aminoethoxy)[2-[octadec-9-enoyloxy]-3-[octadeca-1.11-dien-1-yloxy]propoxy]phosphinic acid | Glycerophosphoethanolamines | -0.36 | 2.02 | 0.04 |
| 17 | 1-(1z-octadecenyl)-2-(9z-octadecenoyl)-sn-glycero-3-phosphoethanolamine | Glycerophosphoethanolamines | -0.51 | 2.54 | 0.04 |
| 18 | 1-(1z-octadecenyl)-sn-glycero-3-phosphocholine | Glycerophosphocholines | -0.45 | 2.93 | 0.01 |
| 19 | 1-palmitoyl-sn-glycero-3-phosphocholine | Glycerophosphocholines | -0.28 | 5.49 | 0.04 |
| 20 | Pc 28:0 | Glycerophosphocholines | 1.25 | 21.08 | 0.04 |
| 21 | Pc 37:4 | Glycerophosphocholines | 0.85 | 2.21 | 0.02 |
| 22 | Pc 42:10 | Glycerophosphocholines | -0.62 | 1.18 | 0.04 |
| 23 | Phosphatidylcholine lyso 20:0 | Glycerophosphocholines | -0.61 | 1.37 | 0.01 |
| 24 | Phosphatidylcholine lyso 20:1 | Glycerophosphocholines | -0.81 | 2.3 | 0 |
| 25 | Phosphatidylcholine lyso alkyl 16:0 | Glycerophosphocholines | -0.47 | 2.14 | 0 |
| 26 | 1-arachidoyl-2-hydroxy-sn-glycero-3-phosphocholine | Glycerophosphocholines | -0.59 | 4.24 | 0.02 |
| 27 | 1-stearoyl-2-myristoyl-sn-glycero-3-phosphocholine | Glycerophosphocholines | -0.75 | 1.17 | 0.03 |
| 28 | Butanoic acid | Fatty acids and conjugates | 0.44 | 2.38 | 0.01 |
| 29 | Octanoic acid | Fatty acids and conjugates | -0.51 | 1.28 | 0.03 |
| 30 | 3h-imidazo(4,5-f)quinoline, 2-amino-3-methyl- | Quinolines | 4.07 | 3.38 | 0.02 |
| 31 | L-carnitine | Quaternary ammonium salts | -0.17 | 19.68 | 0.02 |
| 32 | Acetylcholine | Quaternary ammonium salts | -0.38 | 3.69 | 0.02 |
| 33 | 5-methylcytosine | Pyrimidines and pyrimidine derivatives | -0.57 | 1.68 | 0.01 |
| 34 | Orotate | Pyrimidines and pyrimidine derivatives | 0.71 | 1.6 | 0 |
| 35 | Uridine | Pyrimidine Nucleosides | 0.78 | 4.88 | 0 |
| 36 | Niacinamide | Pyridinecarboxylic acids and derivatives | -0.48 | 10.73 | 0 |
| 37 | Pyridoxal phosphate | Pyridine carboxaldehydes | 0.52 | 2 | 0.02 |
| 38 | Kinetin | Purines and purine derivatives | -0.39 | 1.36 | 0.03 |
| 39 | 3-Methylxanthine | Purines and purine derivatives | 1.13 | 4.33 | 0 |
| 40 | 2-piperidone | Piperidinones | 0.55 | 1.98 | 0.04 |
| 41 | 4-phenylbutyric acid | Phenylbutyrates | -1.24 | 1.48 | 0 |
| 42 | 2-naphthoxyacetic acid | Phenoxyacetic acid derivatives | 0.56 | 4.55 | 0.05 |
| 43 | Taurine | Organosulfonic acids and derivatives | 1.14 | 7.65 | 0.01 |
| 44 | Indolelactic acid | Indolyl carboxylic acids and derivatives | 0.54 | 2.42 | 0.01 |
| 45 | Indole-3-carboxaldehyde | Indoles | 0.59 | 2.7 | 0.02 |
| 46 | 2-ethoxyethanol | Ethers | 0.59 | 1.63 | 0.02 |
| 47 | Enterodiol | Dibenzylbutanediol lignans | 0.32 | 1.22 | 0.03 |
| 48 | Cholesteryl sulfate | Cholestane steroids | 0.39 | 18.12 | 0.04 |
| 49 | N-palmitoyl-d-sphingosine | Ceramides | -1.57 | 1.69 | 0.02 |
| 50 | Propionic acid | Carboxylic acids | 1.56 | 2.29 | 0.01 |
| 51 | 3-hydroxykynurenine | Carbonyl compounds | -0.58 | 2.41 | 0.01 |
| 52 | Etidronic acid | Bisphosphonates | 4.1 | 3.75 | 0.05 |
| 53 | 5-sulfosalicylic acid | Benzenesulfonic acids and derivatives | 0.55 | 9.71 | 0.01 |
| 54 | Homostachydrine | Amino acids, peptides, and analogues | -0.89 | 4.44 | 0.02 |
| 55 | Dl-2,4-diaminobutyric acid | Amino acids, peptides, and analogues | 0.39 | 1.03 | 0.01 |
| 56 | L-pyroglutamic acid | Amino acids, peptides, and analogues | 0.88 | 17.19 | 0.03 |
| 57 | Ng,ng-dimethyl-l-arginine | Amino acids, peptides, and analogues | -0.27 | 3.91 | 0.05 |
| 58 | DL-serine | Amino acids, peptides, and analogues | -0.5 | 1.57 | 0.03 |
| 59 | Phytosphingosine | Amines | -0.08 | 2.5 | 0.02 |
| 60 | 1-methylhistamine | Amines | 1.15 | 2.8 | 0.01 |
| 61 | Paxilline | -- | 1.1 | 3.97 | 0.03 |

-- means the metabolite fails to be assigned to certain class.

**Supplementary table. 2.** **39 vital endogenous metabolites affected by FPT intervention**

| **NO.** | **Metabolites** | **Class** | **FPT/MC** | | |
| --- | --- | --- | --- | --- | --- |
|  |  |  | **Log_2_FC** | **VIP** | ***p*-value** |
| 1 | LysoPC(20:0/0:0) | Glycerophosphocholines | -0.49 | 4.24 | 0.04 |
| 2 | PE(16:0/18:1(9Z)) | Glycerophosphoethanolamines | 0.97 | 3.01 | 0.04 |
| 3 | PS(18:0/18:0) | Glycerophosphoserines | 1.68 | 1.16 | 0.02 |
| 4 | Pg 36:3 | Glycerophosphoglycerols | 0.84 | 2.36 | 0 |
| 5 | Pg 38:6 | Glycerophosphoglycerols | 0.7 | 1.33 | 0.02 |
| 6 | PE(18:0/20:4(5Z,8Z,11Z,14Z)) | Glycerophosphoethanolamines | 0.87 | 11.82 | 0.04 |
| 7 | Pe 34:2 | Glycerophosphoethanolamines | 1.24 | 6.91 | 0.04 |
| 8 | Pe 36:4 | Glycerophosphoethanolamines | 1.27 | 7.33 | 0.02 |
| 9 | Pe 38:6 | Glycerophosphoethanolamines | 1.32 | 9.96 | 0.02 |
| 10 | PC(15:0/15:0) | Glycerophosphocholines | 0.46 | 2.46 | 0.01 |
| 11 | 1-stearoyl-2-linoleoyl-sn-glycero-3-phosphate | Glycerophosphates | -0.81 | 1.22 | 0.03 |
| 12 | Behenic acid | Fatty acids and conjugates | 0.87 | 3.18 | 0 |
| 13 | 12 KETE | Fatty acids and conjugates | 0.73 | 3.52 | 0.02 |
| 14 | Oleic acid | Fatty acids and conjugates | 0.39 | 30.61 | 0.02 |
| 15 | L-palmitoylcarnitine | Fatty acid esters | 1.45 | 7.48 | 0.03 |
| 16 | Oleoyl-l-carnitine | Fatty acid esters | 1.58 | 7.85 | 0.05 |
| 17 | N-[1,3-dihydroxyoctadec-4-en-2-yl]tetracos-15-enamide | Ceramides | -0.68 | 3.98 | 0.05 |
| 18 | N-tetracosenoyl-4-sphingenine | Ceramides | -0.66 | 2.22 | 0.05 |
| 19 | Solanidine | Steroidal alkaloids | 1.55 | 4.51 | 0.03 |
| 20 | .alpha.-ionone | Sesquiterpenoids | -0.91 | 1.99 | 0.02 |
| 21 | Acetylcholine | Quaternary ammonium salts | -0.38 | 4.4 | 0.02 |
| 22 | 5-methylcytosine | Pyrimidines and pyrimidine derivatives | -0.64 | 1.84 | 0.02 |
| 23 | 2'-deoxyuridine 5'-monophosphate | Pyrimidine deoxyribonucleotides | 1.24 | 3.16 | 0.03 |
| 24 | Sphingomyelin (d18:1,18:0) | Phosphosphingolipids | -1.17 | 2.59 | 0.01 |
| 25 | Pratensein | O-methylated isoflavonoids | 0.77 | 1.47 | 0 |
| 26 | Terpinyl acetate | Monoterpenoids | -1.69 | 2.98 | 0.02 |
| 27 | 3-hydroxykynurenine | Carbonyl compounds | -0.41 | 2.17 | 0.03 |
| 28 | Erythritol | Carbohydrates and carbohydrate conjugates | -0.55 | 2.86 | 0.03 |
| 29 | Dapsone | Benzenesulfonyl compounds | -0.27 | 4.41 | 0.04 |
| 30 | *γ*-aminobutyric acid | Amino acids, peptides, and analogues | 1.67 | 1.12 | 0 |
| 31 | Guanidinosuccinic acid | Amino acids, peptides, and analogues | -0.98 | 2.66 | 0.03 |
| 32 | Porphobilinogen | Amines | 0.15 | 1.01 | 0.01 |
| 33 | Quinate | Alcohols and polyols | 1.84 | 2.42 | 0 |
| 34 | (-)-atropine | -- | 1.62 | 2.05 | 0.04 |

-- means the metabolite fails to be assigned to certain class.
